# It’s a long way to the top (if you wanna biplot): a back-to-basics perspective on the implementation of principal component biplots in R

**DOI:** 10.1007/s11135-025-02266-9

**Published:** 2025-07-26

**Authors:** Ettore Settanni, Jagjit Singh Srai

**Affiliations:** Institute for Manufacturing, University of Cambridge, 17 Charles Babbage Road, Cambridge, CB3 0FS UK

**Keywords:** PCA, Biplots, Matrix decomposition, R, Clinical trials supply chains

## Abstract

Principal Component Analysis and biplots are so well-established and readily implemented that it is just too tempting to take for granted their internal workings. In this note we compare how PCA and biplots are implemented in the R language for statistical computing, leveraging a software-agnostic understanding of computational building-blocks that both techniques have in common. We do so with a view to illustrating discrepancies that users might find elusive, as these arise from seemingly innocuous computational choices made under the hood. Wider implications are derived from a simplified case based on real-world clinical trial supply chains data. By getting back to basics, the proposed evaluation grid elevates aspects that are usually disregarded, including relationships that should hold if the computational rationale underpinning each technique is followed correctly. Strikingly, what is expected from these equivalences rarely follows without caveats from the output of specific implementations alone.

## Introduction

A food scientist, a bioinformaticist, and a survey researcher walk into a bar... Jokes aside, what could they possibly have in common? The answer is, probably, PCA—Principal Component Analysis. Many will find the concept familiar, perhaps even trite: PCA is a well-known multivariate analysis technique that has been extensively applied across disciplines—from psychometrics (e.g., Mair 2018) to chemometrics (e.g., Bro and Smilde 2014) as it were—and is now enjoying a renaissance as a staple in data science (see Knox 2018; Marukatat 2023; Gewers et al. 2021).

PCA is often complemented by a biplot, a closely related yet distinct technique whose aim is to jointly visualise the observations and features (variables) that make up a data matrix (Greenacre 2010; le Roux and Gardner 2005; Gower et al. 2011). Some authors (e.g., Abdi and Williams 2010; Mareschal and Brans 1988) describe analogous visualisations without referring to them as biplots.

In principle, biplots and PCA are two sides of the same coin as both are underpinned by matrix decompositions, regardless of specific software used (Rencher & Christensen, 2012, Ch. 12 & 16 Greenacre, 2012). In practice, these techniques are so well-established and readily implemented that their internal workings are simply taken for granted, and approached mechanically. Even in academic work, it is rarely required that computations be disclosed (e.g., Zhang and Ding 2023; Cova et al. 2017). Few resources (Hanson and Harvey 2022) venture into methodological aspects that software users may find elusive, and warn against underplaying their importance.

There is no shortage of options when it comes to implementing PCA and biplots via pre-built software routines. Yet these routines are rarely compared against a software-agnostic understanding based on a common computational structure. In this note we attempt such a comparison, with a view to illustrating discrepancies that may arise from seemingly innocuous computational choices made under the hood.

To keep a reasonable scope, we focus on how PCA and biplots are implemented in R, which is a free, open-source software environment for statistical computing and graphics (R Core Team 2024). Although general-purpose programming languages like Python are more popular amongst developers, R is typically ranked alongside them unlike proprietary alternatives such as STATA and SPSS (see e.g., StackOverflow 2024).

PCA and biplots can be implemented in any of the mentioned languages, and an extensive comparison would be impractical. A reason to focus on R is that its rich ecosystem of ‘packages’ contributed by users facilitates the dissemination of alternative perspectives on specific techniques. Unlike other languages, R contributors are strongly encouraged to take a structured approach to submitting and documenting their shared contributions with a view to benefiting the wider community of adopters (Chambers 2020). Analysis based on such an ecosystem seems appropriate for initiating a conversation about computational aspects that are rarely called into question, but may be implemented differently by routines that users have little incentive to scrutinise.

The remainder of this work is structured as follows. The next section outlines the rationale for selecting specific R packages. Section 3 develops a software-agnostic evaluation grid by reviewing key computational aspects of PCA and biplots. Selected R implementations are then compared in Sect. 4 against the proposed grid with the aid of a simplified example based on real-world clinical trial supply chains data. Possible discrepancies and their practical implications are discussed in Sect. 5 in the light of applications published in research methodology journals. The paper closes with a summary of the contribution and limitations of the proposed approach. To reproduce the analysis that follows, R scripts are provided as Online Supplement (Appendix B).

## Packages selection process

A selection of pre-built PCA and biplots implementations in base-R and contributed R packages is shown in Table 1. Some of these implementations were identified from the literature (e.g., Everitt and Hothorn 2011; Venables and Ripley 2002; Pagès 2014).

**Table 1:** Selected R implementations of PCA and biplots

Group	Package	Functions included		References
		PCA	Biplots	
base-R	stats	prcomp(), princomp()	biplot()	R Core Team (2024)
Generalist	ade4	dudi.pca()	scatter()	Thioulouse et al. (2018)
	amap	acp()	plot()	Lucas (2022)
	FactoMineR	PCA()	plot.PCA()	Lê et al. (2008)
	psych	principal()	biplot.psych()	Revelle (2024)
	vegan	rda()	biplot.rda()	Oksanen et al. (2025)
Specialist, PCA	pcaMethods	pca()	slplot()	Stacklies et al. (2007)
	PCAmixdata	PCAmix()	plot.PCAmix()	Chavent et al. (2022)
	PCAtools	pca()	biplot()	Blighe and Lun (2023)
Specialist, Biplot	biplotEZ	PCA()	PCA.biplot()	Lubbe et al. (2024)
	BiplotGUI		Biplots()	la Grange et al. (2009)
	bpca		bpca()	Faria et al. (2024)
	ggbiplot		ggbiplot()	Vu and Friendly (2024)
	MultBiplotR	PCA.Analysis()	PCA.Biplot()	Vicente-Villardon et al. (2023)

We excluded UBbipl (Gower et al. 2011) which is not distributed on CRAN. Yet several packages follow a similar approach e.g., biplotEZ

Yet comparisons are scant, with few works considering more than one PCA implementation (Hanson and Harvey 2022; Sanchez 2012; Kumar and Paul 2016; Mair 2018; Bakker 2024). Comparisons rarely extend to biplots even though some work reviews software options in R and beyond (Nieto et al. 2014; la Grange et al. 2009).

To address this limitation, we also searched official repositories like CRAN and Bioconductor for keywords such as principal component(s) analysis, PCA and biplots. Overall, we identified 79 packages of potential interest, which can be summarised as follows (a detailed list is provided as Online Supplement, Appendix B):

- 5 generalist packages implementing PCA and biplots in the context of wider subject areas—e.g., ecology, psychometrics, multivariate data analysis.
- 12 biplot-specific packages dedicated to visualisation and user interfaces.
- The remaining are PCA-specific packages, most of which address advanced computational challenges arising e.g., in the field of bioinformatics.

**Fig. 1:**
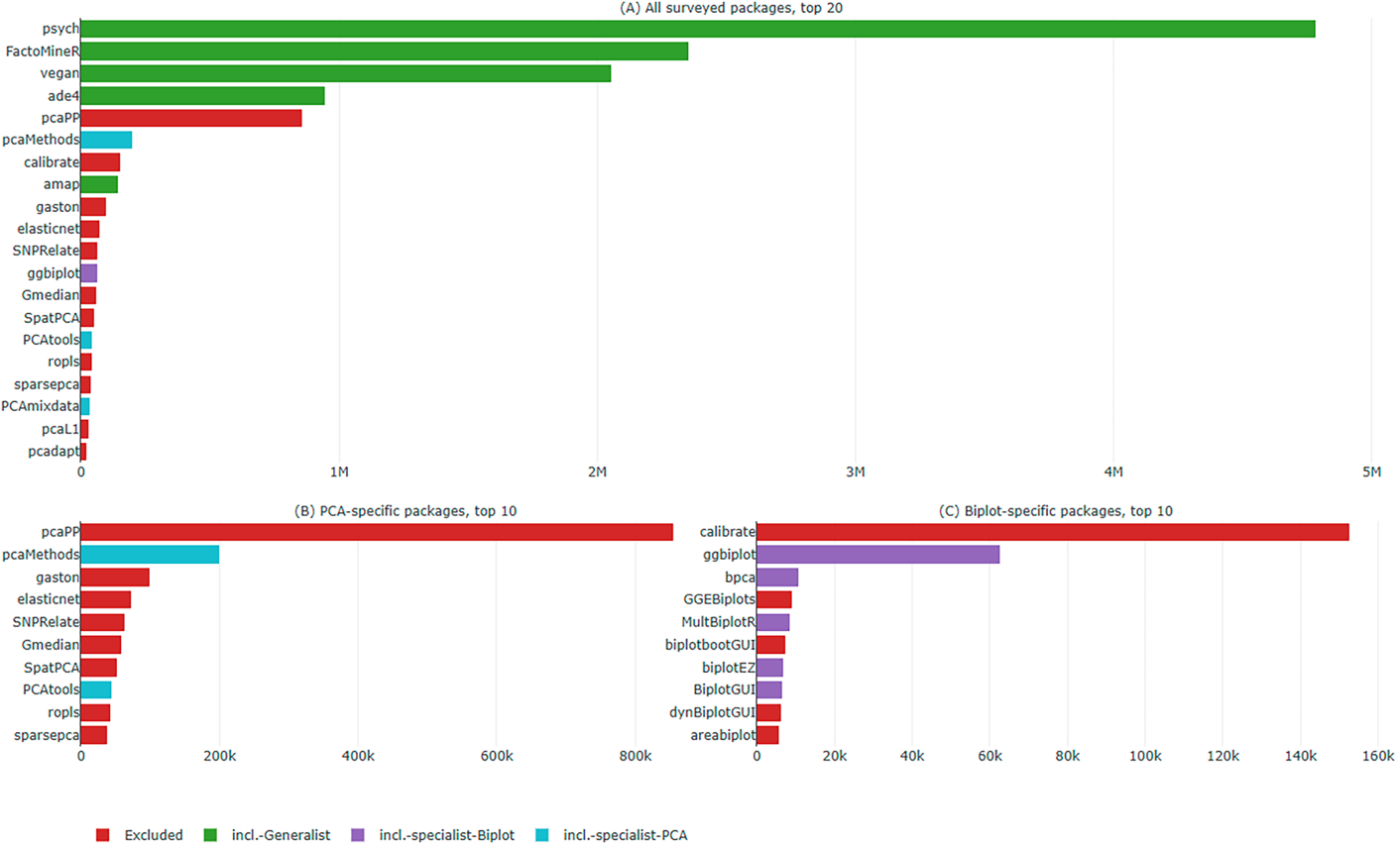
Subset of R packages of interest sorted by downloads from March 2023 to March 2025. Chart created with plotly using data gathered from the package cranlogs and the Bioconductor website

Figure 1 shows a subset of the identified packages ranked by number of downloads over the past two years. The most downloaded packages are generalist, while the most popular specialist packages dedicated to PCA and biplots are well below one million downloads—with the exception of pcaPP (Filzmoser et al. 2024).

Assessing the relevance of specific packages beyond downloads statistics can be challenging. Work providing guidance is nascent (Zeileis et al. 2023; Zhang et al. 2023), and hardly concerned with PCA and biplots specifically. Yet the initial selection had to be narrowed down considerably as this research aims to examine the computations underpinning selected implementations—not to run pre-built code ‘as is’. To achieve that, we excluded packages whose highly specialised computational approaches seemed beyond scope considering the ‘back-to-basics’ perspective adopted in this work (e.g., pcaPP).

The process summarised above led to the subset in Table 1, which is evaluated in Sect. 4 based on the grid outlined next.

## Proposed evaluation grid

Over the past 15 years, several tutorial-like overviews have exemplified key computational aspects of interest for both PCA and biplots (e.g., Gewers et al. 2021; Marukatat 2023; Abdi and Williams 2010; Bro and Smilde 2014; Greenacre 2010). The seasoned practitioner might rightly scoff at the idea of having to endure another one here. Yet it would be misleading to assume there is a single, agreed way to go about these techniques. For example, choosing specific implementations amongst those in Table 1 could imply taking a stance on the conceptual juxtaposition of ‘French’ and ‘North American’ traditions^1^ on multivariate data analysis (see e.g., Holmes 2008).

At the risk of disappointing the advanced reader, this section outlines computational aspects underlying PCA and biplots, with a view to providing a software-agnostic evaluation grid against which specific R implementations can be compared. The less mathematically-oriented reader might be just as disappointed by our detailed derivation of key results included in our grid. Yet this was necessary to avoid borrowing uncritically from the literature. For those willing to engage with the next sub-sections, a simple motivating example is introduced next and used throughout, with R code listings to verify key results. Table 3 in the next section summarises the resulting grid.

### Motivating example

A useful place to start is the visual intuition behind how PCA and biplots work—i.e., that a set of data-points can be represented in a lower-dimensional space while preserving relevant information about them. The analogy with image compression often comes to mind— see e.g., Marukatat (2023); Poole (2014, p. 607).

In the simplest case the data-points are 2-dimensional, as in a data matrix with multiple observations (rows) and just two features or variables (columns)—see e.g., Hanson and Harvey (2022); Abdi and Williams (2010). The task at hand is to determine the data-point’s orthogonal projections onto an appropriately defined line, which yield the desired 1-dimensional representation. While a 2-dimensional setting may appear unrealistic, seldom are more dimensions retained for practical purposes.

**Fig. 2:**
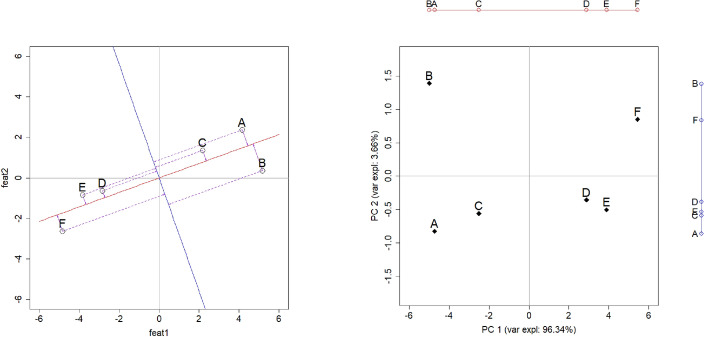
Visualisation of selected information from Table 2. Left: scatterplot of centred data-points corresponding to $\textbf{Y}$ with orthogonal projections on two principal components represented by score vectors $\textbf{z}_{\bullet 1}$ (red) and $\textbf{z}_{\bullet 2}$ (blue); purple arrows represent distances between points and projections. Right: plot of observations using principal component scores as transformed coordinates

The visual intuition described above is shown in Fig. 2 for the fictitious example in Table 2. Before illustrating the process of obtaining Fig. 2 from Table 2, it is worth emphasising that the example is restricted to simple numerical features. Applying PCA to categorical or binary features would require nonlinear generalisations, which pose distinct methodological challenges (see e.g., Atkinson 2024) and are beyond scope.

The inputs and outputs of a typical PCA are summarised in Table 2. The first two columns represent a raw data matrix $\textbf{X} = [x_{ij} ]_{n \times m}$ consisting of $i=1,...,n$ observations and $j=1,...,m$ features, or variables. In this simple example $n=6$ and $m=2$. For each feature *j* the last two rows of Table 2 give its mean $\bar{x}_j=\frac{1}{n}\sum _{i}x_{ij}$, and sample variance $s_{x_j}^{2}=\frac{1}{n-1}\sum _i (x_{ij} - \bar{x}_j)^2$. The columns of Table 2 denoted as $\textbf{Y}$ correspond to the centred data matrix, which is also of size $n \times m$, with generic element $y_{ij} = x_{ij} - \bar{x}_j$. The mean and sample variance for the centred data are also shown in the last two rows of Table 2. For each feature *j* the mean of the corresponding column $\textbf{Y}$ is characteristically zero: $\bar{y}_j=n^{-1}\sum _{i}y_{ij}=\frac{1}{n}\sum _{i}x_{ij}-\bar{x}_j=0$. Yet the sample variance is the same as the raw data’s: $s_{y_j}^{2}=(n-1)^{-1}\sum _i (y_{ij} - \bar{y}_j)^2=\frac{1}{n-1}\sum _i (x_{ij} - \bar{x}_j - 0)^2=s_{x_j}^2$.

Contrary to what many think, standardising the data is not compulsory if the quantities in $\textbf{X}$ are in the same units or scale well (Venables and Ripley 2002, Ch. 11).

**Table 2:** A fictitious example with $m=2$ features and a $n=6$ observations

	Features				Optimal projections, PC1				Optimal projections, PC2			
	Raw $\left[ \textbf{X}\right]$		Centred $\left[ \textbf{Y}\right]$		Proj. coord		Dist of proj. from		Proj. coord		Dist of proj. from	
	Feat1	Feat2	Feat1	Feat2	Feat1	Feat2	Origin $\left[ \textbf{z}_{\bullet 1}\right]$	Observ.	Feat1	Feat2	Origin $\left[ \textbf{z}_{\bullet 2}\right]$	Observ.
A	10.00	6.00	4.17	2.37	4.44	1.59	$-$ 4.72	0.83	$-$ 0.28	0.78	$-$ 0.83	4.72
B	11.00	4.00	5.17	0.37	4.70	1.68	$-$ 4.99	1.39	0.47	$-$ 1.31	1.39	4.99
C	8.00	5.00	2.17	1.37	2.35	0.84	$-$ 2.50	0.56	$-$ 0.19	0.53	$-$ 0.56	2.50
D	3.00	3.00	$-$ 2.83	$-$ 0.63	$-$ 2.71	$-$ 0.97	2.88	0.36	$-$ 0.12	0.34	$-$ 0.36	2.88
E	2.00	2.80	$-$ 3.83	$-$ 0.83	$-$ 3.66	$-$ 1.31	3.89	0.50	$-$ 0.17	0.48	$-$ 0.50	3.89
F	1.00	1.00	$-$ 4.83	$-$ 2.63	$-$ 5.12	$-$ 1.83	5.44	0.85	0.29	$-$ 0.80	0.85	5.44
Mean	5.83	3.63	0.00	0.00	0.00	0.00	0.00	0.75	0.00	0.00	0.00	4.07
Sample var.	18.97	3.13	18.97	3.13	18.88	2.41	21.29	0.14	0.09	0.72	0.81	1.41

This fictitious example is loosely based on Starmer (2018)

Each row of $\textbf{Y}$ is readily interpreted as the coordinates of a point in the Cartesian plane corresponding to one of the $n=6$ observations in Table 2. These coordinates are plotted as shown in Fig. 2 (left). The two lines passing through the origin in the figure are commonly referred to as *principal components* (PCs). The orthogonal projection of a centred data-point onto a given PC is found by intersecting the line representing such component and the perpendicular passing through that point (see e.g., Marecek 2017, Ch. 3). The position of a data-point’s orthogonal projection onto a PC is just its distance from the origin—also referred to as *score*. The sign of a score, however, depends on the quadrant in which the projection falls.

For the example in Table 2, the scores for each PC are collected into two vectors denoted as $\textbf{z}_{\bullet 1}$ (red line) and $\textbf{z}_{\bullet 2}$ (blue line). Fig. 2 (right) shows the plot obtained using the coordinates matrix $$\textbf{Z}=\begin{bmatrix} \textbf{z}_{\bullet 1}&\textbf{z}_{\bullet 2} \end{bmatrix}$$ instead of $\textbf{Y}$: this is commonly referred to as *PCA plot*—not a biplot, yet. How the projections in Fig. 2 (left) are arrived at, and what warrants the claim that they are ‘optimal’, is discussed next.

### PCA-related building blocks

It is worth emphasising that, in the context of PCA, a set of data-points is appropriately represented in a lower-dimensional space when most information about its *variance* is retained. One can imagine rotating the PCs in Fig. 2 (left) until the variance along the scores’ vectors $\textbf{z}_{\bullet 1}$ and $\textbf{z}_{\bullet 2}$ is maximal^2^: the more dispersed the projected data-points along a 1-dimensional PC, the better. Based on Table 2 the variance of the projections onto the first PC (blue line) is about 96% of the projected data-points’ variance:
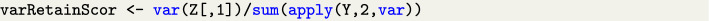


Having established what optimality looks like, the conventional approach to obtain ‘optimal’ PCA scores—attributable to Hotelling (1933)—is to seek appropriate linear combinations of the original data-points’ coordinates (Jolliffe, 1986, Ch. 1; Johnson & Wichern, 1988, Ch. 17). In our example, these coordinates are represented by the columns of the centred data matrix $\textbf{Y}$, and a linear combination of interest is:

1 $$\begin{aligned} \textbf{z} = \textbf{Y}\textbf{a} = \begin{bmatrix} a_1 y_{11} + a_2 y_{12} = z_1\\ \vdots \\ a_1 y_{i1} + a_2 y_{i2} = z_i \\ \vdots \\ a_1 y_{n1} + a_2 y_{n2} = z_n \end{bmatrix} \end{aligned}$$

where $$\textbf{a}=\begin{bmatrix} a_1&a_2 \end{bmatrix}^T$$ is a vector whose elements are the unknown weights of the linear combination, commonly referred to as *loadings*; whereas $\textbf{z}$ may refer to any of the scores vectors introduced in the previous section, whose elements are the distances from the origin for each data-point projection on a given PC.

A distinctive feature of the ‘classical' approach to PCA is the process of finding $\textbf{z}$ in Eq. 1, which revolves around the *sample variance* of its elements. Specifically, if the data has been centred $\textbf{z}$ has mean $\bar{z}=\frac{1}{n}\sum _{i}z_{i}=0$, from which it follows that:

2 $$\begin{aligned} \text {Var} \left( \textbf{z} \right)&= \frac{1}{n-1}\sum _i \left( z_i - \bar{z}\right) ^2 \nonumber \\&= \frac{1}{n-1} \left( \textbf{Ya} - \bar{z}\right) ^T \cdot \left( \textbf{Ya} - \bar{z} \right) \nonumber \\&= \textbf{a}^T\textbf{S}\textbf{a} \end{aligned}$$

where $\textbf{S}=\frac{1}{n-1}\textbf{Y}^T\textbf{Y}$ is the *sample covariance matrix* of $\textbf{Y}$. One must then choose $\textbf{a}$ that maximises $\text {Var}\left( \textbf{z} \right) = \textbf{a}^T\textbf{S}\textbf{a}$ while respecting orthogonality constraints. This is framed as a mathematical program (see e.g., Bro and Smilde 2014; Marukatat 2023):

3 $$\begin{aligned} \max _{\textbf{a}} \quad&\textbf{a}^T\textbf{S}\textbf{a} \nonumber \\ \text {s.t.} \quad&\textbf{a}^T\textbf{a}=1 \end{aligned}$$

As it turns out, solving the program in Eq. 3 for $\textbf{a}$ is equivalent to finding the principal eigenvector of $\textbf{S}$. Indeed, the first-order conditions for a constrained maximum using Lagrange multipliers yields the following identity (Jolliffe, 1986, Ch. 1; Rencher & Christensen 2012, Ch. 12):

4 $$\begin{aligned}&\frac{\text {d}}{\text {d} \textbf{a}}\textbf{a}^T\textbf{S}\textbf{a} - \frac{\text {d}}{\text {d} \textbf{a}} \lambda \left( \textbf{a}^T\textbf{a} -1\right) = 0 \nonumber \\&2\textbf{a}^T\textbf{S} - 2\lambda \textbf{a}^T = 0 \nonumber \\&\left( \textbf{S} - \lambda \textbf{I}\right) \textbf{a} = 0 \end{aligned}$$

which is the familiar eigenvalue problem in linear algebra (e.g., Poole 2014, Ch. 4). Typically, Eq. 4 yields as many eigenvalues and eigenvectors as there are features (Jolliffe 1986, Ch. 1), all of which satisfy the linear combination in Eq. 1.

The building blocks in Eqs. 2–4 may be challenged by more advanced approaches to PCA, including projection pursuit (Croux and Ruiz-Gazen 2005) and lasso regression (Zou et al. 2006)—the former underpins package pcaPP, the most downloaded in Fig. 1B; the latter is implemented by elasticnet. Nonlinear PCA, too, solves a different program than Eq. 3 (e.g., Michailidis and de Leeuw 1998).

Yet, combining Eqs. 4 and 2 yields an important equivalence that is often assumed to hold in practice, regardless of specific software implementations:

5 $$\begin{aligned} \text {Var}\left[ \textbf{z}\right] \quad&= \textbf{a}^T\textbf{S}\textbf{a} \nonumber \\ \quad&= \textbf{a}^T\lambda \textbf{a} \nonumber \\ \quad&= \lambda \end{aligned}$$

#### Remark 1

The variance of a score vector $\textbf{z}$ for a given PC should be identical to the corresponding eigenvalue $\lambda$ of the sample covariance matrix $\textbf{S}$.

Equation 5 is the basis for assessing the decision to retain fewer PCs than the number of features—typically two for graphical display. The quality of such approximation is measured by the relative importance of the two largest eigenvalues of $\textbf{S}$ i.e., the proportion of variance ‘explained’ by the corresponding PCs (Johnson and Wichern 1988, Ch. 17), and is typically reported along the axis of a PCA plot, see Fig. 2 (right).

What is clear from the building blocks summarised so far is that ‘classical' PCA establishes a logical path from seeking a variance-maximising linear combination of the original data-points (Eq. 1) to solving an eigenvalue problem (Eq. 4). In R this is just:
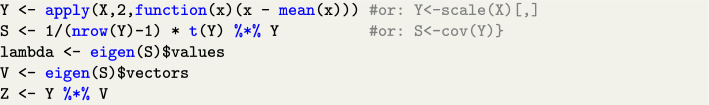


The code begins with centring the data-matrix $\textbf{X}$ and yields the *scores* matrix $\textbf{Z}$, whose columns $\textbf{z}_{\bullet 1}$ and $\textbf{z}_{\bullet 2}$ are shown in Table 2. The *loadings* are the eigenvectors of $\textbf{S}$. Juxtaposing these eigenvectors yields what Gewers et al. (2021) call a ‘transformation’ matrix:

$$\begin{aligned} \textbf{V} = \begin{bmatrix} \textbf{a}_{\bullet 1}&\textbf{a}_{\bullet 2} \end{bmatrix} = \begin{bmatrix} -0.94 & 0.34 \\ -0.34 & -0.94 \end{bmatrix} \end{aligned}$$

with corresponding eigenvalues $$\boldsymbol{\lambda }=\begin{bmatrix} \lambda _1&\lambda _{2} \end{bmatrix}^T= \begin{bmatrix} 21.28&0.81 \end{bmatrix}$$ satisfying Eq. 5:
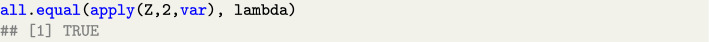


Given the *scores* $\textbf{Z}$ and *loadings* $\textbf{V}$, one can approximate the original data from one or few PCs—a principle widely applied in data compression (see e.g., Marukatat 2023; Gewers et al. 2021). For instance, the R instruction outer(Z[,1], V[,1]) uses the first PC to return the projections’ coordinates shown in Table 2, columns 5-6.

### Biplot-related building blocks

The PCA plot in Fig. 2 may be enriched to become a *biplot*. The difference between these concepts might seem cosmetic: both rely on a ‘transformed’ system of coordinates based on PCA scores, but differ as to what they seek to visualise.

On a deeper level, a biplot is underpinned by a distinct computational strategy: one that yields simultaneously a system of coordinates for both observations and features, so that they can be overlaid in a joint graphical representation. The conventional approach to achieve this reaches back to Gabriel (1971), and is based on the so-called Singular Value Decomposition (SVD) of a data-matrix—see e.g., Gower et al. (2011); Greenacre (2012); le Roux and Gardner (2005); du Toit, Steyn, and Stumpf (1986, Ch. 6).

In practice, doing an SVD of the rectangular data-matrix $\textbf{Y}$ is also the preferred way to obtain the PCA *loadings* and *scores* examined earlier (e.g., Mair, 2018, Ch. 6; Hanson & Harvey, 2022). The standard output of an SVD is (Poole 2014, Ch.7):

6 $$\begin{aligned} \textbf{Y}=\textbf{UD}\textbf{V}^T \end{aligned}$$

where the matrices $\textbf{U}$, $\textbf{V}$ and $\textbf{D}$ are illustrated below applying the command svd(Y) in R to the simple example in Table 2:

- Matrix $$\textbf{U}=\begin{bmatrix}\textbf{u}_{\bullet 1}&\textbf{u}_{\bullet 2}\end{bmatrix}$$ is formed with the ‘left’ eigenvectors of $\textbf{Y}$. In our example:
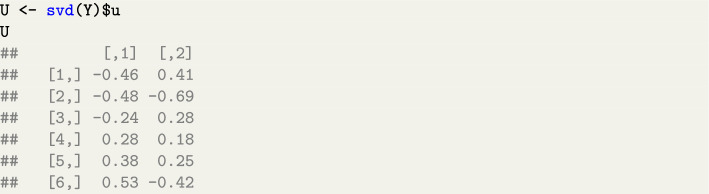
- Matrix $$\textbf{V}=\begin{bmatrix}\textbf{v}_{\bullet 1}&\textbf{v}_{\bullet 2}\end{bmatrix}$$ is formed with the ‘right’ eigenvectors of $\textbf{Y}$, which is analogous to the juxtaposed loadings vectors. In our example:
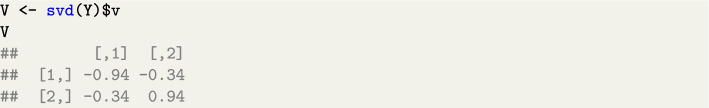
 In Sect. 3.2 matrix $\textbf{V}$ indicates the *loadings* from Eq. 4, which is a slight abuse of notation as the sign of some elements in $$\begin{bmatrix} \textbf{a}_{\bullet 1}&\textbf{a}_{\bullet 2} \end{bmatrix}$$ might differ from $$\begin{bmatrix}\textbf{v}_{\bullet 1}&\textbf{v}_{\bullet 2}\end{bmatrix}$$.
- Vector $$\boldsymbol{\ell }=\begin{bmatrix} l_1&l_2 \end{bmatrix}^T$$ collects the *singular values* of $\textbf{Y}$, and is typically diagonalised to form the matrix $$\textbf{D} = \begin{bmatrix} l_1 & 0 \\ 0 & l_2 \end{bmatrix}$$ in Eq.  6:
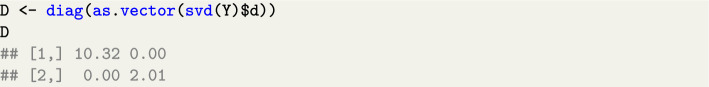

Although SVD is a staple technique, confusion often arises in software implementations about the relationship between singular values and the eigenvalues discussed in Sect. 3.2, which is as follows (Hanson and Harvey 2022; Jolliffe 1986, Ch. 3):

7 $$\begin{aligned} \boldsymbol{\ell } \quad&= (n-1)^{1/2} \begin{bmatrix} \lambda _1^{1/2}&\lambda _2^{1/2} \end{bmatrix}^T \nonumber \\ \quad&= (n-1)^{1/2} \begin{bmatrix} \sqrt{\text {Var}(\textbf{z}_{\bullet 1})}&\sqrt{\text {Var}(\textbf{z}_{\bullet 2})} \end{bmatrix}^T \nonumber \\ \quad&= (n-1)^{1/2} \begin{bmatrix} \sigma _{\textbf{z}_{\bullet 1}}&\sigma _{\textbf{z}_{\bullet 2}} \end{bmatrix}^T \end{aligned}$$

where $\sigma _{\textbf{z}_{\bullet j}}$ is the standard deviation (St. Dev.) of the scores vector $\textbf{z}_{\bullet j}$; and the identity $\lambda _j = \text {Var}[ \textbf{z}_{\bullet j}]$ is based on Eq. 5. One can verify in $\texttt {R}$ that Eq. 7 holds for our example:
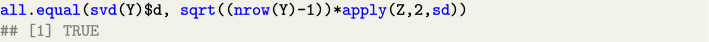


#### Remark 2

The singular values in $\boldsymbol{\ell }$ obtained from an SVD of the data-matrix $\textbf{Y}$ are related to, but distinct from, the eigenvalues $\boldsymbol{\lambda }$ of the *sample covariance matrix* of $\textbf{Y}$.

A biplot is simply an alternative decomposition of the data-matrix $\textbf{Y}$ obtained from Eq. 6 by replacing the diagonal matrix $\textbf{D}$ of singular values with the matrix product $\textbf{D}^{\alpha }\textbf{D}^{1-\alpha }$, whose entries depend on a parameter $0\le \alpha \le 1$ (Ch. 5 Jolliffe 1986). This ‘classic’ biplot decomposition is illustrated below assuming two features:

8 $$\begin{aligned} \textbf{Y}&= \textbf{U}\textbf{D}^{\alpha }\textbf{D}^{1-\alpha }\textbf{V}^T \nonumber \\&= \begin{bmatrix} \textbf{u}_{\bullet 1}&\textbf{u}_{\bullet 2} \end{bmatrix} \begin{bmatrix} l_1 & 0 \\ 0 & l_2 \end{bmatrix}^{\alpha } \begin{bmatrix} l_1 & 0 \\ 0 & l_2 \end{bmatrix}^{1-\alpha } \begin{bmatrix} \textbf{v}_{\bullet 1}^T\\ \textbf{v}_{\bullet 2}^T \end{bmatrix}^{\alpha } \nonumber \\&= \begin{bmatrix} u_{11}l_1^{\alpha } & u_{12}l_2^{\alpha } \nonumber \\ u_{21}l_1^{\alpha } & u_{22}l_2^{\alpha } \nonumber \\ \vdots & \vdots \nonumber \\ u_{n1}l_1^{\alpha } & u_{n2}l_2^{\alpha } \nonumber \\ \end{bmatrix} \begin{bmatrix} v_{11}l_1^{(1-\alpha )} & v_{21}l_1^{(1-\alpha )} \nonumber \\ v_{12}l_2^{(1-\alpha )} & v_{22}l_2^{(1-\alpha )} \nonumber \\ \end{bmatrix} \nonumber \\&= \textbf{A}\textbf{B}^T \end{aligned}$$

In the presence of more than two features, the first two columns of $\textbf{A}$ and rows of $\textbf{B}^T$ are retained, and Eq. 8 becomes a rank-2 approximation of $\textbf{Y}$:

9 $$\begin{aligned} \textbf{Y} \approx \textbf{Y}_{(2)} = \textbf{A}\textbf{B}^T \end{aligned}$$

A *Principal Component biplot* (PC biplot) is a specific type of biplot obtained from Eq. 8 when $\alpha = 0$ (Venables and Ripley 2002, Ch. 11; Gabriel 1971). Some R packages use the term ‘GH plot’ instead, introduced by work like Nieto et al. (2014).

For a PC biplot, the matrices $\textbf{A}$ and $\textbf{B}^T$ in Eq. 8 take on the following meaning:

- $\textbf{A}=\textbf{U}\textbf{D}^0=\textbf{U}$ provides the coordinates of each observation (listed row-wise);
- $\textbf{B}^T=\textbf{D}\textbf{V}^T$ gives the coordinates of each feature (listed column-wise).

A feature of the PC biplot is that the matrix of observations’ coordinates $\textbf{A}$ is a scaled version of its counterpart in a PCA plot. With a slight abuse of notation, right-multiplying both sides of Eq. 6 by the *loadings* matrix $\textbf{V}$ yields:

10 $$\begin{aligned} \textbf{YV}&= \textbf{UD}\textbf{V}^T \textbf{V} \nonumber \\ \textbf{Z}&= \textbf{UD} \end{aligned}$$

although, in practice, the sign of some *scores* in $\textbf{Z}$ might differ from those in Sect. 3.2. Since $\textbf{A} = \textbf{U}$ when $\alpha =0$, it follows from Eq. 10 that the axes of a PC biplot are those of a PCA plot divided by the corresponding singular values:

11 $$\begin{aligned} \textbf{A}&= \textbf{Z}\textbf{D}^{-1} \nonumber \\&=\begin{bmatrix}\textbf{z}_{\bullet 1}l_1^{-1}&\textbf{z}_{\bullet 2}l_2^{-1}\end{bmatrix} \end{aligned}$$

Some references disagree with Eq. 11 (e.g., Gewers et al. 2021). Yet it is promptly verified in R for our simple example—with the caveat that it only holds in absolute values when $\textbf{Z}$ is computed as shown in Sect. 3.2 rather than Eq. 10:
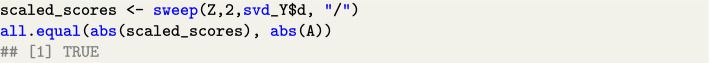


Another characteristic of a PC biplot is that the features coordinates $\textbf{B}^T$ are a scaled version of the *loadings*. For later use, these coordinates can be re-expressed in terms of the St.Dev. of the *scores* based on Eqs. 7-8:

12 $$\begin{aligned} \textbf{B}^T&= \textbf{D}\textbf{V}^T \nonumber \\&= \begin{bmatrix}\textbf{v}_{\bullet 1}^T l_1 \\ \textbf{v}_{\bullet 2}^T l_2\end{bmatrix} \nonumber \\&= (n-1)^{1/2}\begin{bmatrix} \textbf{v}_{\bullet 1}^T \sigma _{\textbf{z}_{\bullet 1}} \\ \textbf{v}_{\bullet 2}^T \sigma _{\textbf{z}_{\bullet 2}} \end{bmatrix} \end{aligned}$$

**Fig. 3:**
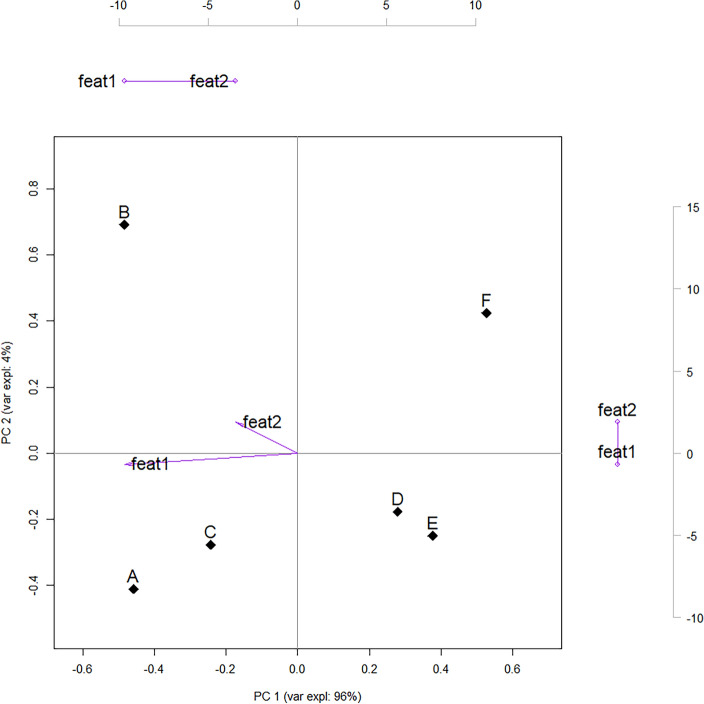
PC biplot for the fictitious example in Table 1 based on Eq. 11, 12. The superposed dual axes (coloured grey) reflect the different scale of the features’ coordinates (purple-coloured arrows). To make it comparable with Fig. 2 (right) the *score*s matrix is as described in Sect. 3.2

In our example, the observations’ coordinates $\textbf{A}=\textbf{U}$ were given at the beginning of this section, whereas Eq. 12 yields the following features coordinates matrix $\textbf{B}^T$:
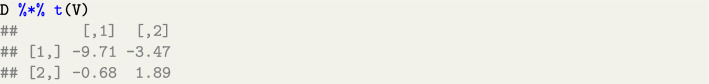


A ‘conventional’ PC biplot with these values from Eq. 11, 12 is shown in Fig. 3. Choosing $\alpha =1$ in Eq. 8 would have yielded a different set of coordinates: the observations would be those of a PCA plot (Sect. 3.2) i.e. $\textbf{A}= \textbf{Z}$ due to Eq. 10; and the *loadings* would serve as features’ coordinates i.e., $\textbf{B}^T=\textbf{V}^T$—a representation often used in practice for features (e.g., Field et al. 2012, Ch.17).

#### Remark 3

In a biplot the coordinates of observations and features are jointly computed from the parametrised matrix decomposition in Eq. 8. Overlaying a plot of loadings and a plot of PCA scores, whilst still a biplot, is not a PC biplot.

It is a matter of debate whether the superposed Cartesian axes in Fig. 3 provide a suitable representation of the features in a biplot. An alternative is to represent features as calibrated axes that extend through the diagram instead of vectors or arrows (Gower et al. 2011, Ch. 2 & 3; le Roux and Gardner 2005). In this way, one should be able to project an observation point onto a calibrated biplot axis and read off the values of the corresponding feature. This graphical representation is adopted in practice by several R implementations, including calibrate (Graffelman and van Eeuwijk 2005)—the most downloaded package in Fig. 1(C).

Unlike a PC biplot, the ‘calibrated biplot axes’ approach described above requires that the coordinates for observations and features be obtained from Eq. 8 choosing $\alpha = 1$. As it turns out, this choice is necessary for calibrating the biplot axes as described in (Gower et al. 2011, Ch. 2)—Appendix A summarises key underlying computations. Replicating the graphical display of calibrated biplot axes in full, however, is beyond scope as dedicated packages (e.g., biplotGUI) provide useful interfaces.

What is clear is that the chosen parametrisation of Eq. 8 affects the admissible interpretations of the geometrical representations obtained (Jolliffe 1986, Ch. 5; Venables and Ripley 2002, Ch. 11). For PC biplots, these aspects will be discussed next.

### Building blocks linked to geometrical properties

Features and observations are represented in a biplot as vectors of coordinates whose properties are often of practical interest for the analyst. In this subsection we examine a subset of four such properties—three concerning features—which are often mentioned but rarely accompanied by a proof (e.g., Nieto et al. 2014).

A common assumption is that the length of the vector formed by the coordinates of a feature *j* equals the St. Dev. $\sigma _{\textbf{y}_{\bullet j}}$ of the corresponding column $\textbf{y}_{\bullet j}$ of the data-matrix $\textbf{Y}$ (du Toit et al. 1986, p. 108; Venables and Ripley 2002, p. 312;). Yet this assumption might not hold automatically. To show why this is so, we pre-multiply both sides of Eq. 8 by $\textbf{Y}^T$ (Jolliffe 1986, p. 77), and assume $\alpha =0$:

13 $$\begin{aligned} \textbf{Y}^T\textbf{Y}&= \textbf{Y}^T\textbf{A}\textbf{B}^T \nonumber \\&= \textbf{B}\textbf{U}^T\textbf{U}\textbf{B}^T \nonumber \\ (n-1)\textbf{S}&= \textbf{B}\textbf{B}^T \end{aligned}$$

where $\textbf{U}^T\textbf{U}=\textbf{I}$ since the columns of $\textbf{U}$ are orthonormal. For a generic diagonal element $s_{jj}$ of $\textbf{S}$, and corresponding row of $\textbf{B}$, denoted as $\textbf{b}_{j \bullet }$ Eq. 13 becomes:

14 $$\begin{aligned} (n-1)s_{jj} \quad&= \textbf{b}_{j \bullet }^T \textbf{b}_{j \bullet } \nonumber \\ \sigma _{\textbf{y}_{\bullet j}} \quad&= (n-1)^{-\frac{1}{2}} \left\Vert \textbf{b}_{j \bullet } \right\Vert _2 \nonumber \\ \quad&= \sqrt{\frac{1}{n-1}\sum _k\left( v_{jk}l_k\right) ^2} \nonumber \\ \quad&= \sqrt{\sum _k \left( v_{jk} \sqrt{\lambda _k} \right) ^2} \end{aligned}$$

where summation is over $k=1,\ldots , m$ PCs; and $l_j = \left( {n-1} \right) ^{1/2} \sqrt{\lambda _j}$ is based on Eq. 7.

In R one can verify the second line of Eq. 14 for all features *j* as follows:
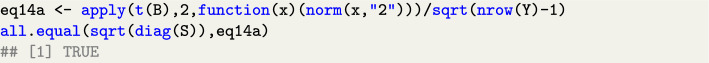


Eq. 14 suggests that the length of the vector representing a given feature in a PC biplot is proportional, not equal to the feature’s Std. Dev.; for an identity to hold the feature’s coordinates should be computed using the eigenvalues of the covariance matrix $\textbf{S}$, instead of the singular values of $\textbf{Y}$ (e.g., Everitt and Hothorn 2011, p. 92). Another way to impose such an identity is to multiply the observation’s coordinates $\textbf{A}$ by $(n-1)^{1/2}$ and the feature’s coordinates $\textbf{B}$ by $(n-1)^{-1/2}$ (Jolliffe 1986, p. 78).

The relationship between a feature’s Std. Dev. and the length of its coordinates vector in Eq. 14 assumes a PC biplot and might not hold if $\alpha = 1$ is chosen.

#### Remark 4

If a biplot is a PC biplot then, given a feature *j*, its St. Dev. $\sigma _{\textbf{y}_{\bullet j}}$ is proportional, but not identical, to the length of vector $\textbf{b}_{j \bullet }$ representing its coordinates. In the presence of more than two features, such relationship is only an approximation.

Another property that biplots are expected to exhibit is that the cosine of the angle between two vectors of features’ coordinates is identical to the correlation between these features (e.g., Everitt and Hothorn 2011, p. 93; du Toit et al. 1986, p. 108). Next we show that for this property to hold, the features’ coordinates must be computed according to Eq. 8. As a corollary we also show that this relationship is just an approximation for 2-dimensional representations (Gabriel 1971).

We start by observing that, just like Eq. 14, the coordinates of a given feature *j* are collected in a vector $\textbf{b}_{j\bullet }$—the *j*-th row of $\textbf{B}$ in Eq. 12. Given a pair of features $j=1,2$ squaring the 2-norm of their difference $\left\Vert \textbf{b}_{1\bullet } - \textbf{b}_{2\bullet } \right\Vert _2^2$ leads to the following ‘textbook’ identity (for a proof see Binmore and Davies 2001, p. 18):

15 $$\begin{aligned} \cos \theta _{1,2} \quad&= \frac{\langle {\textbf{b}_{1\bullet }, \textbf{b}_{2\bullet }}\rangle }{\left\Vert \textbf{b}_{1\bullet } \right\Vert _2 \cdot \left\Vert \textbf{b}_{2\bullet } \right\Vert _2} \end{aligned}$$

which is the definition of *cosine similarity*. In Eq. 15 the angle in radians between the features vectors is denoted by $\theta _{1,2}$, and $\langle {\cdot ,\cdot } \rangle$ is the inner product between vectors.

The other identity of interest is the Pearson product-moment correlation between the same features, obtained from the relevant columns of the data-matrix $\textbf{Y}$:

16 $$\begin{aligned} \text {corr}_{\textbf{y}_{\bullet 1},\textbf{y}_{\bullet 2}} = \frac{\langle {\textbf{y}_{\bullet 1}, \textbf{y}_{\bullet 2}}\rangle }{\left\Vert \textbf{y}_{\bullet 1}\right\Vert _2 \cdot \left\Vert \textbf{y}_{\bullet 2}\right\Vert _2} \end{aligned}$$

#### Proposition 1

$\text {corr}_{\textbf{y}_{\bullet 1},\textbf{y}_{\bullet 2}} = \cos \theta _{1,2}$ (the Pearson product-moment correlation between two columns of the centred data-matrix $\textbf{Y}$ is identical to the cosine distance between the corresponding columns of the features coordinates matrix $\textbf{B}^T$).

#### Proof

Let $\textbf{u}_{i\bullet }$ denote the *i*-th row of matrix $\textbf{A}$. Recalling Eqs. 11-12, the numerator in Eq. 16 can be re-stated as follows:

17 $$\begin{aligned} \langle {\textbf{y}_{\bullet 1}, \textbf{y}_{\bullet 2}}\rangle \quad&= \langle { \begin{bmatrix} \textbf{u}_{1\bullet } \textbf{b}_{1\bullet }&\dots&\textbf{u}_{i\bullet } \textbf{b}_{1\bullet }&\dots&\textbf{u}_{n\bullet } \textbf{b}_{1\bullet } \end{bmatrix}, \begin{bmatrix} \textbf{u}_{1\bullet } \textbf{b}_{2\bullet }&\dots&\textbf{u}_{i\bullet } \textbf{b}_{2\bullet }&\dots&\textbf{u}_{n\bullet } \textbf{b}_{2\bullet } \end{bmatrix}}\rangle \nonumber \\ \quad&=\left( \textbf{U}\textbf{b}_{1\bullet }\right) ^T \textbf{U}\textbf{b}_{2\bullet } \end{aligned}$$

Equation 17 also affects the denominator of Eq. 16 since $\left\Vert \textbf{y}_{\bullet j}\right\Vert _2 = \sqrt{\langle {\textbf{y}_{\bullet j}, \textbf{y}_{\bullet j}}\rangle }$. Substituting Eq. 17 in Eq. 16, and recalling that $\textbf{U}^T \textbf{U}=\textbf{I}$, one obtains:

18 $$\begin{aligned} \frac{\langle {\textbf{y}_{\bullet 1}, \textbf{y}_{\bullet 2}}\rangle }{\left\Vert \textbf{y}_{\bullet 1}\right\Vert _2 \cdot \left\Vert \textbf{y}_{\bullet 2}\right\Vert _2} \quad&= \frac{\textbf{b}_{1\bullet }^T\textbf{U}^T \textbf{U}\textbf{b}_{2\bullet }}{\sqrt{\textbf{b}_{1\bullet }^T\textbf{U}^T \textbf{U}\textbf{b}_{1\bullet }}\sqrt{\textbf{b}_{2\bullet }^T\textbf{U}^T \textbf{U}\textbf{b}_{2\bullet }}} \nonumber \\ \quad&= \frac{\textbf{b}_{1\bullet }^T\textbf{b}_{2\bullet }}{\sqrt{\textbf{b}_{1\bullet }^T\textbf{b}_{1\bullet }}\sqrt{\textbf{b}_{2\bullet }^T\textbf{b}_{2\bullet }}} \nonumber \\ \quad&= \frac{\langle {\textbf{b}_{1\bullet }, \textbf{b}_{2\bullet }}\rangle }{\left\Vert \textbf{b}_{1\bullet }\right\Vert _2 \cdot \left\Vert \textbf{b}_{2\bullet }\right\Vert _2} \nonumber \\ \text {corr}_{\textbf{y}_{\bullet 1},\textbf{y}_{\bullet 2}} \quad&= \cos \theta _{1,2} \end{aligned}$$

which completes the proof of the equivalence between Eq. 15 and Eq. 16$\square$

#### Remark 5

The identity in Eq. 18 assumes a PC biplot for which all dimensions have been retained in the decomposition in Eq. 8, and may not hold for rank-2 approximations (Eq. 9).

Our simple example conveniently includes only two features: the cosine similarity between $\textbf{b}_{1\bullet }$ and $\textbf{b}_{2\bullet }$ is $\cos \theta _{1,2}=0.84$, and its equivalence to the correlation coefficient between $\textbf{y}_{\bullet 1}$ and $\textbf{y}_{\bullet 2}$ is promptly verified in R:
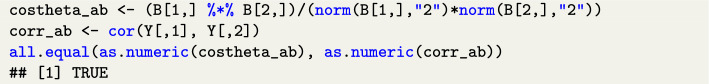


In addition to the correlation between features, practitioners are often interested in how features correlate with PCs. Specifically, the *loading* of a feature onto a PC is often thought of as equivalent to the correlation between them, thus providing a measure of the substantive importance of such feature to that PC (e.g., Field et al. 2012, Ch. 17). Indeed, *loadings* serve as features’ coordinates in a biplot when the parametrisation $\alpha =1$ is chosen in Eq. 8. Yet confusions arise in practice about the correct relationship between features’ coordinates and correlation coefficients.

(Johnson and Wichern 1988, Ch. 8) show that the generic element $v_{jk}$ in a loading matrix $\textbf{V}$ is an intuitive measure of the importance of the *j*-th feature for the *k*-th PC, and that such loading is proportional—not identical—to the correlation coefficients between the corresponding PC scores vector $\textbf{z}_{\bullet k}$ and feature vector $\textbf{y}_{\bullet j}$:

#### Proposition 2

(Johnson and Wichern, 1988) the correlation coefficient between the *k*-th PC score $z_{\bullet k}$ and the *j*-th feature $y_{\bullet j}$ is proportional to $v_{jk}$, the *j*-th element of the *k*-th loading:

19 $$\begin{aligned} \text {corr}_{\textbf{z}_{\bullet k},\textbf{y}_{\bullet j}}=\frac{v_{jk}\sqrt{\lambda _k}}{\sqrt{s_{jj}}} \end{aligned}$$

where $s_{jj}$ is the *j*-th diagonal element of the sample covariance matrix $\textbf{S}$ and $\lambda _k$ is its *k*-th eigenvalue (see Sect. 3.2).

#### Proof

From Eq. 16 we obtain the identity:

$$\begin{aligned} \text {corr}_{\textbf{z}_{\bullet k},\textbf{y}_{\bullet j}} = \frac{\langle {\textbf{z}_{\bullet k}, \textbf{y}_{\bullet j}}\rangle }{\left\Vert \textbf{z}_{\bullet k}\right\Vert _2 \cdot \left\Vert \textbf{y}_{\bullet j}\right\Vert _2} \end{aligned}$$

Starting from the denominator, we invoke Eq. 2 and Eq. 5 and find that $\left\Vert \textbf{z}_{\bullet k}\right\Vert _2= \sqrt{(n-1)\text {Var}(\textbf{z}_{\bullet k})}=\sqrt{(n-1)}\sqrt{\lambda _k}$. The same reasoning yields $\left\Vert \textbf{y}_{\bullet k}\right\Vert _2=\sqrt{(n-1)\text {Var}(\textbf{y}_{\bullet j})}=\sigma _{y_{\bullet j}}\sqrt{(n-1)}$. Moving on to the numerator, from Eq. 1 we have $\textbf{z}_{\bullet k}=\textbf{Y}\textbf{v}_{\bullet k}$ (the notation for the loadings is the one in Sect. 3.3). We define a vector $\textbf{e}_j$ of appropriate size having 1 in *j*-th position and 0 elsewhere, so that $\textbf{y}_{\bullet j}=\textbf{Y}\textbf{e}_j$. Invoking Eq. 2 and Eq. 4 we get: $\langle {\textbf{z}_{\bullet k}, \textbf{y}_{\bullet j}}\rangle =(n-1)\textbf{v}_{\bullet k}^T\textbf{S}\textbf{e}_j=(n-1)\lambda _k\textbf{v}_{\bullet k}^T\textbf{e}_j=(n-1){v}_{jk}$. Substituting:

20 $$\begin{aligned} \frac{\langle {\textbf{z}_{\bullet k}, \textbf{y}_{\bullet j}}\rangle }{\left\Vert \textbf{z}_{\bullet k}\right\Vert _2 \cdot \left\Vert \textbf{y}_{\bullet j}\right\Vert _2}&=\frac{(n-1)\lambda _k{v}_{jk}}{(n-1)\sigma _{y_{\bullet j}}\sqrt{\lambda _k}} \end{aligned}$$

from which one obtains Eq. 19$\square$

In our example Eq. 19 holds for any feature-score combination e.g., $j=1,k=2$:
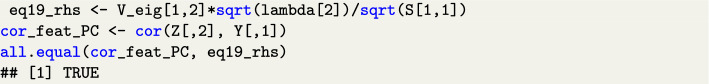


#### Remark 6

Comparing the right-hand side of Eq. 19 with Eq. 14 suggests that the features’ coordinates of a PC biplot contain the information needed to evaluate $\text {corr}_{\textbf{z}_{\bullet k},\textbf{y}_{\bullet j}}$.

So far we have focussed on properties concerning the representation of features in a PC biplot. To close the section we examine a well-known relationship concerning the squared Euclidean distance between two generic rows *h* and *i* of the observations’ coordinates matrix $\textbf{A}=\textbf{U}$ in a PC biplot. Such distance is the squared 2-norm $d_{hi}^2 = \left\Vert \textbf{u}_{h\bullet }-\textbf{u}_{i\bullet }\right\Vert _2^2$ and is proportional to the squared Mahalanobis distance between the *h*-th row and the *i*-th row of the data-matrix $\textbf{Y}$ (for a proof: Jolliffe 1986, Ch. 5):

21 $$\begin{aligned} (\textbf{y}_{h \bullet }-\textbf{y}_{i \bullet })^T\textbf{S}^{-1}(\textbf{y}_{h \bullet }-\textbf{y}_{i \bullet }) =(n-1)d_{hi}^2 \end{aligned}$$

where $\textbf{S}^{-1}$ is the inverse of the sample covariance matrix of $\textbf{Y}$, if it exists. In our example, Eq. 21 holds for any observations pair e.g. $h=1, i=2$:
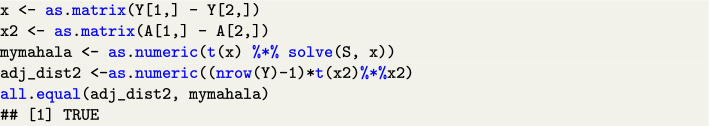


#### Remark 7

In a PC biplot the distances between observations are such that less weight is given to features with larger variance and to groups of highly correlated features. Eq. 21 is only approximated if two PCs are retained in the presence of more than two features (Eq. 9).

## Comparative insights

In this section, we compare the R implementations selected in Sect. 2 against the computational building blocks reviewed in Sect. 3 and summarised in Table 3, which serve as a software-agnostic evaluation grid. Comparative insights are provided upfront in Tables 4, 5 and 6, where the evaluation criteria are shown as column headers. The following subsections detail how these insights came about with the aid of a simplified example based on clinical trial supply chains data, which is introduced next.

### Extended example: clinical trial supply chains data

The fictitious example in Sect. 3.1 served the purpose of replicating detailed analytical results for each computational building block. Yet such an example might be perceived as having little practical relevance, and too simplistic for deriving wider implications from the proposed comparison. Here we outline a more concrete example, albeit simplified and desensitised, based on medicines shortage data in clinical trials.

#### Context and data overview

A Clinical Trial Supply Chain (CTSC) delivers investigational medicinal products (IMP) and is characterised by higher service levels compared to a commercial pharmaceutical supply chain. Higher penalties are typically associated with shortfalls i.e., events that prevent patients enrolled in clinical trials from getting the right IMP when required by the study protocol (Ch. 14 Mills 2024). Such risk is typically managed in CTSCs through increased inventory, although greater attention is paid to trade-offs with the associated waste from write-offs ( Settanni et al. 2018).

For commercial medicines, shortages may be reported by a regulatory agency under specific conditions, but the collection and analysis of this data is a challenge (Geyman et al. 2020). For pharmaceutical manufacturers service levels are sensitive data, although self-reported figures are occasional surveyed and benchmarked against managerial practices (e.g., Friedli et al. 2013)

The illustrative example in Table 7 is a streamlined, desensitised excerpt from data collected by a major manufacturer on 50+ trials that experienced at least one shortage between 08/2013 and 08/2024. The original excerpt shared with the authors was generated by querying the manufacturer’s Randomization and Trial Supply Management (RTSM) technology platform—a digital infrastructure providing transaction records across the ‘last mile’ nodes of a CTSC (Mills 2024, Ch.6).

In the context of this work data is meant to be illustrative, and limited to 11 protocols (clinical trials) and 4 numerical features for tractability: (1) count of stockout events (STO); (2) n. of patients enrolled (ENR); (3) n. of clinics (CLI); and (4) trial duration in months (DUR). For visualisation, we also provide a nominal variable for the protocol’s therapeutic area (THA), and a binary variable indicating whether the underlying active pharmaceutical ingredient is biological rather than chemical (BIO).

**Fig. 4:**
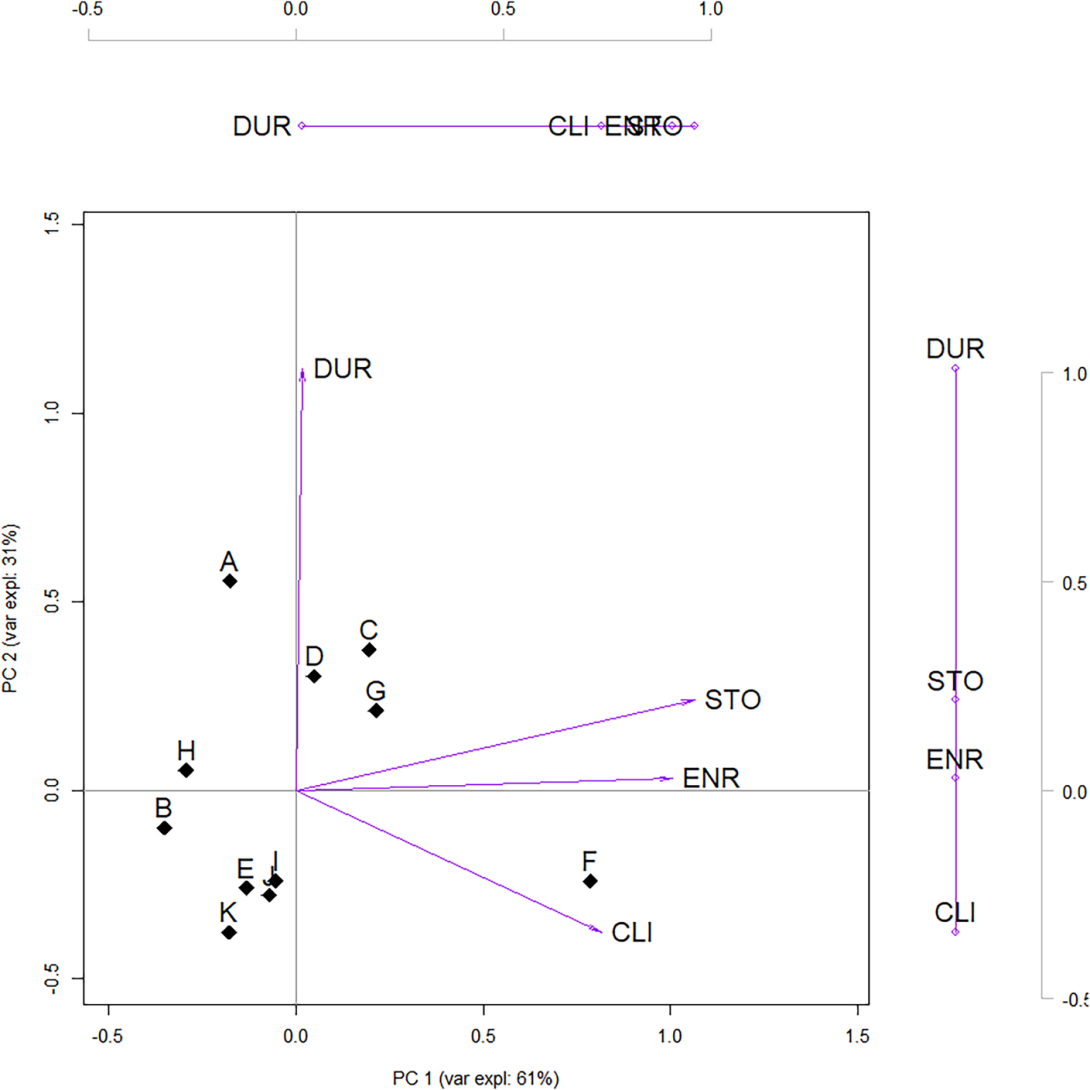
Biplot of observations and features (shown as numbers) for an extended example based on SVD. The superposed dual axes outside the plot reflect the different scale of the features’ coordinates

Our software-agnostic approach (Sect. 3 and Table 3) is applied first. Fig. 4 shows the resulting PC biplot whose coordinates can be read in Tables 7 and 8. Table 8 also shows how features and PC scores correlate, whereas cosine similarities and correlations between features represented in our PC biplot are shown in Table 9.

**Table 3:** Summary of proposed evaluation grid: computational building blocks, associated remarks and propositions (proofs)

Building blocks		Decomposition			Equations			Remarks	
		eigen$(\textbf{S})$	svd$\,\,\,\,(\textbf{Y})=\textbf{U}\textbf{D}\textbf{V}^T$		n.	Main relationship	Propos’n w/proof	n.	Summary
			$\textbf{D}$	$\textbf{D}^{\alpha }\textbf{D}^{1-\alpha }$					
I.A	Eigenvectors (loadings)	$\bullet$			4	$\textbf{V}=\left[ a_{jk}\right] _{m\times m}$			
I.B	Eigenvalues (ev)	$\bullet$			4	$\boldsymbol{\lambda }=\left[ \lambda _k \right] _{m \times 1}$			
I.C	PCA scores	$\bullet$			1	$\textbf{Z}=\textbf{Y}\textbf{V}$			
I.D	Scores variance	$\bullet$			5	$\text {Var} \left[ \textbf{z}_{\bullet k} \right] = \lambda _k$		1	Score’s variance for *k*-th PC identical to corresponding ev.
II.A	Right eigenvectors (loadings)		$\bullet$		6	$\textbf{V}=\left[ v_{jk}\right] _{m\times m}$ and $\left| {v}_{jk}\right| =\left| a_{jk}\right|$			
II.B	singular values (sv)				6	$\boldsymbol{\ell }=\left[ \ell _k\right] _{m \times 1}$			
II.C	PCA scores (SVD)		$\bullet$		10	$\textbf{Z}=\textbf{U}\textbf{D}$			
II.D	relationship between sv and ev		$\bullet$		7	$(n-1)^{-\frac{1}{2}}\ell _k=\sqrt{\lambda _k}$		2	sv related to, but distinct from ev.
III.A	Observations coord.			$\alpha =0$	11	$\textbf{A}=\textbf{U}=\textbf{Z}\textbf{D}^{-1}$		3	Coordinates jointly computed: geometrical interpretations PC biplot and “scores and loadings” plot differ
III.B		$\bullet$		$\alpha =1$	8	$\textbf{A}=\textbf{Z}$			
IV.A	Features coord.			$\alpha =0$	12	$\textbf{B}^T=\textbf{D}\textbf{V}^T$			
IV.B		$\bullet$		$\alpha =1$	8	$\textbf{B}=\textbf{V}$			
V.A	Feat. St.Dev.			$\alpha =0$	14	$(n-1)^{-\frac{1}{2}}\left\Vert \textbf{b}_{j \bullet } \right\Vert _2 =\sigma _{y_{\bullet j}}$		4	Proportional, not identical to the feat coord vector length. only approx for rank-2
VI.A	Feat. correl.			$\alpha =0$	18	$\text {corr}_{\textbf{y}_{\bullet i},\textbf{y}_{\bullet j}} = \text {cos}\left( \theta _{ij}\right)$	1	5	Holds if all dimensions retained, not for rank-2 approx
VI.B	Feat.-score correl.		$\bullet$		19	$\text {corr}_{\textbf{z}_{\bullet k},\textbf{y}_{\bullet j}}=\frac{v_{jk}\sqrt{\lambda _k}}{\sqrt{s_{jj}}}$	2	6	Proportional, not equal to loading. PC biplot’s feat coord contain relevant info.
VII.A	Observations dist.			$\alpha =0$	21	$(n-1)d_{hi}^2=(\textbf{y}_{h \bullet }-\textbf{y}_{i \bullet })^T\textbf{S}^{-1}(\textbf{y}_{h \bullet }-\textbf{y}_{i \bullet })$		7	Dist. between observ. in PC biplot weights less features with large var, high corr.

**Table 4:** Assessment of selected implementations in R, with a focus on *PCA*. Column headers: evaluation grid in Table 3. Symbols indicate if pre-built outputs match the software-agnostics results in Table 7 exactly ($\checkmark$) or in absolute value ($\circ$), and the presence of discrepancies ($\times$). Text refers to the function’s outputs returning specific results to the user, or to results that are produced internally but invisible to users (“int.”)

Package	Function	Decomposition		Eigenvectors (loadings)		Eigen/singular values		Scores	
		Type	Matrix	(I.A)	(II.A)	(I.B)	(II.B)	(I.C)	(II.C)
				$$\textbf{V}=\left[ a_{jk}\right]$$	$$\textbf{V}=\left[ v_{jk}\right]$$	$$\boldsymbol{\lambda }=\left[ \lambda _k \right]$$	$$\boldsymbol{\ell }=\left[ \ell _k \right]$$	$$\textbf{Z}=\textbf{Y}\textbf{V}$$	$$\textbf{Z}=\textbf{U}\textbf{D}$$
*base*-R									
stats	prcomp	svd	$$\textbf{Y}$$		$$\checkmark$$ rotation		$$\checkmark$$ (int.)	$$\checkmark$$ x	
	princomp	eigen	$$\textbf{Y}^T\textbf{Y}\frac{1}{n}$$	$$\circ$$ loadings$$^{1}$$		$$\times$$ (int.)		$$\circ$$ scores$$^{1}$$	
*Generalist*									
ade4	dudi.pca	eigen	$$\textbf{Y}^T\textbf{Y}\frac{1}{n}$$	$$\circ$$ c1$$^{1}$$		$$\times$$ eig	$$\times$$ (int.)	$$\circ$$ li$$^{1}$$	
amap	acp	eigen	$$\textbf{Y}^T\textbf{Y}$$	$$\circ$$ loadings$$^{1}$$			$$\checkmark$$ eig$$^{1}$$	$$\circ$$ scores$$^{1}$$	
FactoMineR	PCA	svd	$$\textbf{Y}\frac{1}{\sqrt{n}}$$		$$\circ$$ svd$V	$$\times$$ eig	$$\times$$ svd$vs$$^{1}$$		$$\circ$$ ind$coord
psych	principal	eigen	$$\textbf{S}$$	$$\times$$ loadings$$^{3}$$		$$\checkmark$$ values		$$\times$$ scores$$^{3}$$	
vegan	rda	svd	$$\textbf{Y}\frac{1}{\sqrt{n-1}}$$		$$\checkmark$$ CA$v	$$\checkmark$$ CA$eig	$$\times$$ (int.)	$$\times$$ scores()$$^{2}$$	
*Specialist, PCA*									
pcaMethods	pca	svd	$$\textbf{Y}$$		$$\checkmark$$ loadings		$$\checkmark$$ (int.)	$$\checkmark$$ scores	
PCAmixdata	PCAmix	svd	$$\textbf{Y}\frac{\sqrt{n}}{\sqrt{n-1}}$$		$$\times$$ V	$$\times$$ eig	$$\checkmark$$ (int)	$$\times$$ scores	$$\times$$ ind$coord
PCAtools	pca	svd	$$\textbf{Y}$$		$$\checkmark$$ loadings		$$\checkmark$$ (int.)		$$\checkmark$$ rotated
*Specialist, biplots*									
biplotEZ	PCA	svd	$$\textbf{Y}$$		$$\checkmark$$ Vr	$$\times$$ eigenvalues	$$\times$$ (int.)	$$\checkmark$$ (int.)$$^{5}$$	
BiplotGUI	Biplots	eigen	$$\textbf{Y}^T\textbf{Y}$$	$$\circ$$ (int.)		$$\times$$ (int)	$$\times$$ (int)	$$\circ$$ (int.)	
bpca	bpca	svd	$$\textbf{Y}$$		$$\checkmark$$ eigenvectors	$$\times$$ eigenvalues	$$\checkmark$$ (int.)		$$\checkmark$$ (int.)$$^{5}$$
ggbiplot	getsvd$$^{6}$$	svd	$$\textbf{Y}$$		$$\checkmark$$ V		$$\times$$ D		$$\times$$ (int.)
MultiBiplotR	PCA.Analysis	svd	$$\textbf{Y}$$		$$\checkmark$$ EV	$$\times$$ EigenValues	$$\checkmark$$ (int.)		$$\checkmark$$ (int.)$$^{5}$$

$^1$ Imprecisions in relationship between eigenvalues and singular values and/or in chosen covariance matrix (for PCAs based on eigen);

$^2$ scores() is a function taking the result of rda as input. The output sites is equivalent to multiplying each column of $\textbf{Z}$ by a constant.;

$^3$ Discrepancy due to default internal scaling: the *k*-th column of the scores (loadings) matrix is divided (multiplied) by $\sqrt{\lambda _k}$;

$^5$ PCA scores can only be obtained indirectly as observations coordinates when a scores and loadings biplot option is selected;

$^6$ Assumes SVD but requires results from other functions e.g. prcomp, princomp and dudi.pca

**Table 5:** Assessment of R implementations, with a focus on *biplots*. Column headers: evaluation grid in Table 3. Symbols indicate if pre-built outputs match the software-agnostics results in Tables 8, 9 exactly ($\checkmark$) or in absolute value ($\circ$), and the presence of discrepancies ($\times$). Text refers to the function’s outputs returning specific results to the user, or to results that are produced internally but invisible to users (“int.”)

Package	Function	Biplot coordinates					Calibr. axes$^{2}$
		PC biplot ($\alpha =0$)		Scores and loadings biplot ($\alpha =1$)		$\alpha$ arg.$^{1}$	
		(III.A)	(IV.A)	(III.B)	(IV.B)		
		$\textbf{A}=\textbf{Z}\textbf{D}^{-1}$	$\textbf{B}^T=\textbf{D}\textbf{V}^T$	$\textbf{A}=\textbf{Z}$	$\textbf{B}=\textbf{V}$		
*base*-R							
stats	biplot$^{4}$						
	.prcomp	$\times$ (int.)$^{5}$	$\times$ (int.)$^{5}$	$\checkmark$ (int.)	$\checkmark$ (int.)	scale	
	.princomp	$\circ$ (int.)$^{5}$	$\circ$ (int.)$^{5}$	$\circ$ (int.)$^{5}$	$\circ$ (int.)$^{5}$	scale	
*Generalist*							
ade4	scatter$^{6}$	$\times$ c0$^{5,6}$	$\times$ l1$^{5,6}$	$\circ$ c1$^{5,6}$	$\circ$ li$^{5,6}$	Permute	
amap	biplot.acp			$\circ$ scores$^{5,6}$	$\circ$ loadings$^{5,6}$		
FactoMineR	plot.PCA		$$\times$$ var$coord	$$\times$$ ind$coord			
psych	biplot.psych		$\times$ loadings$^{6}$	$\times$ scores$^{6}$			
vegan	biplot.rda	$\times$ sites$^{7}$	$\times$ species$^{7}$				
*Specialist, PCA*							
pcaMethods	slplot$^{6}$			$\checkmark$scores	$\checkmark$ loadings		
PCAmixdata	plot.PCAmix		$$\times$$ quanti$coord	$$\times$$ ind$coord			
PCAtools	biplot$^{6}$			$\checkmark$ rotated	$\checkmark$ loadings		
*Specialist, biplots*							
biplotEZ	PCA.biplot$^{4}$	$\times$ Z	$\times$ Lmat	$\checkmark$ Z	$\checkmark$ Lmat	correlation. biplot	$\checkmark$
BiplotGUI	Biplots	$\times$ (int.)	$\times$ (int.)	$\circ$ (int.)	$\circ$ (int.)	(menu)	$\checkmark$
bpca	plot	$$\times$$ coord$objects$$^{8}$$	$$\times$$ coord$variables$$^{8}$$	$$\checkmark$$ coord$objects	$$\checkmark$$ coord$variables	method	
ggbiplot	ggbiplot$$^{4}$$	$$\times$$ plot_env$df.u	$$\times$$ plot_env$df.v	$$\times$$ plot_env$df.u	$$\times$$ plot_env$df.v	scale	
MultBiplotR	PCA.Biplot	$\times$ $^{8}$ RowCoordinates	$\times$ $^{8}$ ColCoordinates	$\times$ $^{8}$ RowCoordinates	$\times$ $^{8}$ ColCoordinates	alpha	

$^1$ Argument equivalent to $\alpha$ switching between PC biplot and scores and loadings biplot;

$^2$ Calibrated biplot axis approach (Sect. 3.3 and Appendix A);

$^3$ Results from base-R functions prcomp and princomp accepted as input;

$^5$ Imprecisions in relationship between eigenvalues and singular values or in chosen covariance matrix (for PCAs based on eigen); or issues carried over from Table 4;

$^6$ Biplot coordinates returned by corresponding PCA function and passed on as argument;

$^7$ Coordinates proportional to expected results;

$^8$ Due to further, non-optional scaling

**Table 6:** Verification of key identities for PCA and PC biplots. Symbols denote whether both an identity holds ($\checkmark$) or not ($\times$). Some discrepancies are discussed in footnotes. Identities V.A, VI.A and VII are not applicable (n.a.) to “scores and loadings biplots”, and require that all PCs are retained when verifying them

Package	Function	PCA decomposition		Function	Biplot geometric			
		(II.B)$^{1}$	(I.D)$^{1}$		(V.A)$^{2}$	(VI.A)	(VI.B)$^{2}$	(VII)$^{2}$
		$\sqrt{\lambda _k}=\frac{\ell _k}{\sqrt{n-1}}$	$\text {Var}\left[ \textbf{z}_k \right] =\lambda _k$		$\sigma _{y_{\bullet j}}=\frac{1}{\sqrt{n-1}}\left\Vert \textbf{b}_{j \bullet } \right\Vert _2$	$\text {corr}_{\textbf{y}_{\bullet i},\textbf{y}_{\bullet j}} = \text {cos}\left( \theta _{ij}\right)$	$\text {corr}_{\textbf{z}_{\bullet k},\textbf{y}_{\bullet j}}=\sigma _{y_{\bullet j}}^{-1}v_{jk}\sqrt{\lambda _k}$	
*base*-R								
stats				biplot				
	prcomp	$\checkmark$ sdev	$\checkmark$	.prcomp	$\times$ $^{3}$	$\checkmark$	$\checkmark$	$\times$ $^{3}$
	princomp	$\times$ sdev$^{3}$	$\times$	.princomp	$\times$ $^{4}$	$\checkmark$	$\times$ $^{4}$	$\times$ $^{3}$
*Generalist*								
ade4	dudi.pca	$\times$ dval (int.)$^{3}$	$\times$	scatter	$\times$ $^{3,4}$	$\checkmark$	$\times$ $^{4}$	$\checkmark$ $^{5}$
amap	acp	$\times$ sdev, eig$^{3}$	$\times$	biplot.acp	(n.a.)	(n.a.)	$\times$ $^{4}$	(n.a.)
FactoMineR	PCA	$$\times$$ svd$vs	$$\times$$	plot.PCA	$$\times$$	$$\checkmark$$	$$\times$$ var$cor	(n.a.)
psych	principal	$\checkmark$	$\times$	biplot.psych	$\times$	$\checkmark$	$\times$	$\checkmark$ $^{5}$
vegan	rda	$\times$	$\times$	biplot.rda	$\times$	$\checkmark$	$\checkmark$	$\times$
*Specialist, PCA*								
pcaMethods	pca	$\checkmark$ sDev	$\checkmark$	slplot	(n.a.)	(n.a.)	$\checkmark$	(n.a.)
PCAmixdata	PCAmix	$\checkmark$	$\times$	plot.PCAmix	$\times$	$\checkmark$	$\times$	$\times$
PCAtools	pca	$\checkmark$ sdev	$\checkmark$	biplot	(n.a.)	(n.a.)	$\checkmark$	(n.a.)
*Specialist, biplots*								
biplotEZ	PCA	$\times$	$\times$	PCA.biplot	$\times$	$\times$	$\times$ $^{4}$	$\times$
BiplotGUI	Biplots	$\times$	$\times$	Biplots	$\times$	$\checkmark$	$\times$ $^{4}$	$\times$
bpca	bpca	$\times$	$\times$	plot	$\times$	$\checkmark$	$\checkmark$ var.rb	$\checkmark$ $^{5}$
ggbiplot	getsvd	$\times$ $^{6}$	$\times$ $^{6}$	ggbiplot	$\times$	$\checkmark$	$\checkmark$ $^{6}$	$\times$
MultBiplotR	PCA.Analysis	$\times$	$\times$	PCA.Biplot	$\times$	$\checkmark$	$\times$ Structure	$\times$

$^1$ For functions based on SVD (eigendecomposition), the singular values (eigenvalues) are those produced internally and the eigenvalues (singular values) are computed as shown in Sect. 3;

$^2$ Requires a sample covariance matrix $\textbf{S}$, and assumes $\sigma _{\textbf{y}_{\bullet ,j}} = \sqrt{s_{jj}}$: for eigen-based PCAs $\textbf{S}$ is the one decomposed in Table ?? but S=cov(Y) for svd-based PCAs;

$^3$ The identity may hold if $(n-1)$ is replaced by a function-specific value e.g., *n*;

$^4$ $\sigma _{\textbf{y}_{\bullet ,j}} \ne \sqrt{s_{jj}}$ but the identity holds if $\sigma _{\textbf{y}_{\bullet ,j}}$ is replaced by $\sqrt{s_{jj}}$;

$^5$ Mahalanobis distance identical, not proportional to Euclidean;

$^6$ Based on function-specific computations. The relationship might hold for the chosen PCA input

**Table 7:** PCA scores and observations’ coordinats on biplot for extended case example with $m=4$ features and $n=11$ observations. Computations as in Sect. 3.3

Protocol ID	Raw data $\textbf{X}$ $^{1}$				Centred data $\textbf{Y}$				PCA scores$^{2}$ $\textbf{Z}_{\text {SVD}}=\textbf{UD}$				Biplot$^{3}$ $\textbf{A}=\textbf{U}$	
	STO	ENR	CLI	DUR	STO	ENR	CLI	DUR	PC1	PC2	PC3	PC4	PC1	PC2
A	0.12	0.23	0.10	1.00	$-$ 0.11	$-$ 0.17	$-$ 0.20	0.61	$-$ 0.26	0.60	0.00	$-$ 0.15	$-$ 0.17	0.56
B	0.00	0.00	0.00	0.23	$-$ 0.23	$-$ 0.40	$-$ 0.29	$-$ 0.15	$-$ 0.53	$-$ 0.11	$-$ 0.14	0.11	$-$ 0.35	$-$ 0.10
C	0.65	0.49	0.24	0.71	0.42	0.09	$-$ 0.05	0.33	0.30	0.41	$-$ 0.16	0.11	0.20	0.37
D	0.29	0.48	0.26	0.71	0.06	0.08	$-$ 0.03	0.33	0.07	0.33	0.05	$-$ 0.05	0.05	0.30
E	0.00	0.30	0.32	0.14	$-$ 0.23	$-$ 0.11	0.03	$-$ 0.24	$-$ 0.20	$-$ 0.28	0.04	$-$ 0.05	$-$ 0.13	$-$ 0.26
F	1.00	1.00	1.00	0.16	0.77	0.60	0.71	$-$ 0.22	1.19	$-$ 0.26	$-$ 0.13	$-$ 0.04	0.79	$-$ 0.24
G	0.35	0.83	0.26	0.58	0.12	0.43	$-$ 0.03	0.20	0.33	0.23	0.27	0.10	0.22	0.21
H	0.06	0.05	0.04	0.41	$-$ 0.17	$-$ 0.35	$-$ 0.26	0.02	$-$ 0.44	0.06	$-$ 0.13	0.05	$-$ 0.29	0.05
I	0.00	0.53	0.26	0.14	$-$ 0.23	0.13	$-$ 0.03	$-$ 0.25	$-$ 0.08	$-$ 0.26	0.23	0.05	$-$ 0.05	$-$ 0.24
J	0.06	0.30	0.43	0.14	$-$ 0.17	$-$ 0.10	0.14	$-$ 0.24	$-$ 0.11	$-$ 0.30	$-$ 0.01	$-$ 0.12	$-$ 0.07	$-$ 0.28
K	0.00	0.20	0.30	0.00	$-$ 0.23	$-$ 0.20	0.00	$-$ 0.39	$-$ 0.27	$-$ 0.41	$-$ 0.04	$-$ 0.00	$-$ 0.18	$-$ 0.38
Mean	0.23	0.40	0.29	0.38	$-$ 0.00	$-$ 0.00	$-$ 0.00	0.00	$-$ 0.00	0.00	$-$ 0.00	0.00	0.00	$-$ 0.00
Sample var.	0.11	0.09	0.07	0.10	0.11	0.09	0.07	0.10	0.23	0.12	0.02	0.01	0.10	0.10

$^1$ Due to sensitivity, data was transformed by unitisation (see e.g., la Grange et al. 2009, Appendix);

$^2$ Scores matrix based on Eq. 10;

$^3$ Approximated observations’ coordinates on a 2-dimensional PC biplot from Eq. 11

**Table 8:** Loadings and feature’ coordinates on biplot for extended case example with $m=4$ features and $n=11$ observations. Computations as in Sect. 3.3. Roman numbers refer to building blocks in Table 3

	Loadings				PC biplot coordinates				Features properties				
	$\textbf{V}$ (I.A)				$\textbf{B}$ (IV.A)		$\left\Vert \textbf{b}_{j \bullet } \right\Vert _2$ (V.A)		$\sigma _{\textbf{y}_{\bullet j}}=\sqrt{s_{jj}}$	Corr. w/PCs (VI.B)			
	PC1	PC2	PC3	PC4	PC1	PC2	rank-2	all PCs		PC1	PC2	PC3	PC4
*Feature*													
STO	0.64	0.20	$-$ 0.62	0.41	0.96	0.22	0.99	1.03	0.33	0.93	0.21	$-$ 0.28	0.11
ENR	0.60	0.03	0.77	0.22	0.91	0.03	0.91	0.98	0.31	0.93	0.03	0.36	0.07
CLI	0.49	$-$ 0.31	$-$ 0.14	$-$ 0.80	0.74	$-$ 0.34	0.81	0.85	0.27	0.87	$-$ 0.40	$-$ 0.07	$-$ 0.27
DUR	0.01	0.93	0.07	$-$ 0.37	0.02	1.01	1.01	1.02	0.32	0.02	0.99	0.03	$-$ 0.10
*Eigenvalues and singular values (II.B)*													
$\boldsymbol{\lambda }=\left[ \lambda _k\right]$	0.23	0.12	0.02	0.01									
$\boldsymbol{\ell }=\left[ \ell _k\right]$	1.51	1.09	0.46	0.29									

Notation used: $\left\Vert \textbf{b}_{j \bullet } \right\Vert _2$ is the length of the vector of biplot coordinates of the *j*-th feature (i.e., the *j*-th row of $\textbf{B}$); $\sigma _{\textbf{y}_{\bullet j}}$ is the corresponding feature’s St.Dev. in the data-matrix $\textbf{Y}$, which is identical to the *j*-th element $s_{jj}$ on the diagonal of its covariance matrix $\textbf{S}$; $^{1}$ $\textbf{V}$ obtained by SVD of matrix $\textbf{Y}$ as in Eq. 6. Eigenvectors obtained by eigendecomposition (II.A) are equivalent in absolute values but some signs may change; $^{2}$The features’ coordinates matrix $\textbf{B}$ is as defined in Eq. 12; $^{3}$The biplot is obtained from the rank-2 approximation as in Eq.9; $^{4}$If all dimensions are retained, then Eq. 14 holds: $\sigma _{\textbf{y}_{\bullet j}}=(n-1)^{-\frac{1}{2}}\left\Vert \textbf{b}_{j \bullet } \right\Vert _2$

**Table 9:** Features correlations and angle between features’ vectors in a PC biplot (VI.A in Table 3)

Feature pair		$\textrm{cos}(\theta _{a,b})$		Correlation	
a	b	Rank-2	all PCs	$\textrm{corr}_{\textbf{y}_{\bullet a},\textbf{y}_{\bullet b}}$	*p*-value
STO	ENR	0.98	0.78	0.78	0.0048
STO	CLI	0.79	0.72	0.72	0.0132
STO	DUR	0.24	0.2	0.2	0.5481
ENR	CLI	0.89	0.75	0.75	0.0074
ENR	DUR	0.05	0.05	0.05	0.8885
CLI	DUR	− 0.4	− 0.36	− 0.36	0.277

#### Insights from PC biplot representation

The results introduced above are based on desensitised data, and are meant primarily to be an aid for methodological comparison. With this caveat in mind, a brief commentary on these results seems appropriate given their practical relevance.

The first thing worth noting is that the 2-dimensional representation of the 11 clinical trials, displayed as points in Fig. 4, is an adequate approximation since the variance explained by the two PCs retained is more than 92%. Eyeball inspection and the correlations in Table 8 and  9 suggest the following interpretation for the arrangement of observations (points) and features (arrows) along the two PCs. The horizontal dimension seems to denote operational complexity—a combination of size of the network of clinics served (i.e., patients enrolled and number of sites), and challenges encountered in supplying to such a network (i.e., shortfalls). Arrangements along the vertical dimension are almost exclusively representative of a protocol's duration—a feature with poor or no association with the others.

Based on this understanding, protocol F—a Phase IIIA respiratory trial that experienced most stock-outs—stands out in the bottom-right quadrant of Fig. 4 as it exhibits a unique combination of operational complexity in demand and supply, and relatively short duration. Protocols D, C and G—a cluster of specialist infectious disease trials—occupy the upper-right quadrant: they experienced fewer stock-outs than F, but more than other protocols, and exhibit longer durations. Proceeding counterclockwise one encounters protocols A, B, H—the only phase II trials in the dataset—which exhibit medium-high duration and lower complexity. In the bottom right corner protocols E, J and K form a cluster of high-priority, respiratory trials involving large molecules (biologics). Protocol I is very close to J in terms of Mahalanobis distance despite sharing none of the cluster’s nominal features.

Next, we apply the 15 pre-built R implementations identified in Table 1 to the same numerical example, and elaborate on the extent to which our software-agnostic results are matched (Tables 4, 5, 6). Detailed results and graphical displays are omitted, but can be reproduced with the scripts provided in the Online Supplement (Appendix B).

### Group 1: SVD-based PCA approaches

About 20% of the implementations reviewed are underpinned by the decomposition coded as svd$(\textbf{Y})$ in Table 4. The analysis is centred on Eqs. 6 and 8, with the base-R function svd often at work under the hood.

In base-R, the joint deployment of prcomp and biplot covers the building-blocks discussed in Sect. 3.3. For example, prcomp(X, scale = FALSE) takes the raw data-matrix $\textbf{X}$ as a direct input, and returns a matrix of scores $\textbf{Z}$ (Eq. 10) and loadings $\textbf{V}$ (Eq. 6) that match exactly the results in Tables 7 and 8. Yet issues arise when transforming scores and loadings into PC biplot coordinates. The singular values vector $\boldsymbol{\ell }$ is often computed in the background but not returned as an output (see Table 4). Imprecisions in representing the relationships between singular values, eigenvalues and PCA scores are not uncommon, and seldom are PC biplot coordinates returned that match exactly those suggested by a software-agnostic approach (see Table 5).

For instance, prcomp returns $(n-1)^{-\frac{1}{2}}\boldsymbol{\ell }$ as sdev, which suggests that the following assumptions are made: (1) the *k*-th singular value $\ell _k$ is identical to $\sqrt{\lambda _k}$ by virtue of Eq. 7, and (2) the latter is identical to the St.Dev. of the PCA scores by virtue of Eq. 5. Several implementations generate a similar output (see Table 6), suggesting that the building blocks I.D and II.D in Table 3 are assumed to hold, but rarely computed.

The above has implications for functions like biplot and ggbiplot, as these build on results from prcomp—as well as other implementations—to generate the coordinates of a PC biplot. In the specific case of biplot, results from the underlying computations are not returned to users. Inspection of the script stats:::biplot.prcomp suggests that Eqs. 11, 12 are correctly implemented under the hood, except that the diagonal matrix of singular values $\textbf{D}$ does not satisfy Eq. 7 due to $\sqrt{n}$ being used instead of $\sqrt{n-1}$. Table 6 shows that this apparently innocuous choice affects some identities that should hold if the coordinates of a PC biplot are correctly determined.

Similar considerations apply to curated R packages in this group, including PcaMethods (Stacklies et al. 2007) and PCAtools (Blighe and Lun 2023). Both are meant primarily for bioinformatics applications and offer a homonym function pca. When SVD is chosen among several methods, both libraries rely directly or indirectly on base-R capabilities, producing scores and loadings identical to prcomp.

Unlike base-R, the parametrisation $\alpha =1$ (Eq. 8) is implicit in functions like pcaMethods::slplot and PCAtools::biplot, whose outputs are plots of scores and loadings—either juxtaposed or overlaid—with coordinates $\textbf{A}=\textbf{Z}$ and $\textbf{B}=\textbf{V}$. Much like stats::biplot these results are not returned to the user, but can be inferred by inspection of the underpinning R scripts. In PCAtools::biplot the loadings are scaled ‘cosmetically’ to improve the look of the display: whilst this practice is not unusual, one feature in our example would not appear in the plot due to arbitrarily set bounds.

### Group 2: SVD-based biplot approaches

Nearly all the specialised biplots packages reviewed (about 27% of total) rely on SVD, as one would expect from the classic approach discussed in Sect. 3.3. Compared to implementations in the previous section, those in this group provide more refined graphical interfaces. Yet when it comes to PC biplots, none of them fully matches the results expected from a software-agnostic approach (see Table 5 and 6).

In most cases, an SVD is done internally via dedicated functions (see Table 4). An exception is the ggbiplot package (Vu and Friendly 2024), which requires that an SVD be done separately, and sets out to reconcile inputs from alternative PCA implementations through the function $\texttt {get\_SVD}$. Yet the proposed reconciliation framework has some shortcomings, and the resulting PC biplot coordinates disagree with Eqs. 11, 12.

For instance, assume that prcomp—which was examined earlier—provides initial inputs. Inspection of the script ggbiplot::get_SVD shows that the familiar SVD notation of Eq. 6 is slightly abused: in this context, $\textbf{D}$ becomes a diagonal matrix whose elements are the singular values divided by $\sqrt{n-1}$ i.e., sdev from prcomp; and instead of the left eigenvectors matrix one gets $\textbf{U}=\textbf{Z}\left[ \textbf{D}\times \sqrt{n}\right] ^{-1}$. With these inputs, ggbiplot generates the following coordinates based on Eq. 8: $\textbf{A}=\textbf{U}\textbf{D}^{1-\gamma }$ and $\textbf{B}=\psi \textbf{V}\textbf{D}^\gamma$, where $\gamma =1$ yields a PC biplot and $\psi$ represents further scaling beyond the classic building blocks examined so far. This approach is conducive to the discrepancies observed in Tables 4, 5, affecting several properties in Table 6.

Similarly to ggbiplot, also the specialist package biplotEZ (Lubbe et al. 2024) disagrees with the original meaning of $\textbf{D}$ in Eq. 6 when computing the coordinates of a PC biplot. Unlike ggbiplot, $\textbf{D}$ is replaced by a diagonal matrix $\mathbf {\Delta }$ whose elements are the square roots of the singular values of $\textbf{Y}^T\textbf{Y}$ divided by $\sqrt{n-1}$, in sharp disagreement with Eq. 7. The coordinates returned by biplotEZ for a PC biplot become $\textbf{A}=\textbf{Z}\mathbf {\Delta }^{-1}$ and $\textbf{B}=\textbf{V}\mathbf {\Delta }^{-1}$ causing a major departure from Eqs. 11, 12.

An additional aspect of biplotEZ is that it features calibrated biplot axes in the tradition of Gower et al. (2011). For reasons discussed in Appendix A, the axes calibration process typically assumes a “scores and loadings” biplot (i.e., it assumes $\alpha =0$ in Eq. 8). Even though biplotEZ extends the process to PC biplots, the underlying computations may be affected by the discrepancies discussed above.

The remaining two packages in this group are bpca and MultiBiplotR. In both cases the departure from the classic approach to PC biplots is less drastic than in the previous ones. The approach followed by MultiBiplotR (Vicente-Villardon et al. 2023) is similar to ggbiplot’s with important differences: (1) $\textbf{D}$ is correctly interpreted; and (2) both coordinates’ matrices are further scaled as follows: $\textbf{A}=\psi \textbf{U}\textbf{D}^{1-\gamma }$ and $\textbf{B}=\frac{1}{\psi }\textbf{V}\textbf{D}^\gamma$, where $\gamma =1$ yields a PC biplot, and the scalar $\psi$ is consistent with the concept of “lambda scaling” discussed in Gower et al. (2011, Ch.2). Unfortunately such scaling is not optional, causing most of the discrepancies observed for this approach.

The bpca package (Faria et al. 2024) is an overall correct implementation of the classic matrix decomposition in Eq. 8. However, it imposes additional scaling on the biplot coordinates matrices, following a process originally devised by Gabriel (1971) to transform building blocks V and VII (Table 3) from proportional relationships to identities (see Jolliffe 1986, Ch. 5). Like MultiBiplotR, discrepancies with the proposed grid could have been addressed had such scaling been optional.

One last aspect of the implementations in this group is that outputs designated as “eigenvalues”, if present, are without exception a misnomer for $\boldsymbol{\ell }^2$ (see Eq. 7).

### Group 4: eigen-decomposition

One third of the reviewed implementations follow more closely the ‘original’ concept of PCA described in Sect. 3.2. Most of them belong to the ‘generalist’ type discussed in Sect. 2 and differ in which matrix constitutes the input for an eigendecomposition. Table 4 shows it rarely is $\textbf{S}$, the covariance matrix of $\textbf{Y}$ as assumed in our grid.

The base-R function princomp—nearly a homonym of prcomp—follows Eq. 4 closely. Yet the underlying definition of covariance matrix disagrees with Eq. 2 as it considers the number of observations *n* instead of $$n-1$$. From Table 4 it is clear that this choice does not affect the scores, so long as the function is executed with arguments princomp(X, fix_sign = T). Yet the covariance matrix differs from $$\textbf{S}$$, and the eigenvalues returned by squaring the output princomp()$sdev do not satisfy Eq. 5.

The matrices of biplot coordinates in Eqs. 11, 12 may be obtained from an eigendecomposition, so long as the relationship between singular values and eigenvalues in Eq. 7 holds. The base-R function biplot discussed earlier accepts inputs from princomp. In principle, the combination of princomp and biplot violates Eq. 7, since Eq. 5 no longer holds. Yet the combination of incorrect assumptions in princomp and biplot produces the ‘correct’ matrices $\textbf{A}$ and $\textbf{B}$.

Curated packages that implement an eigendecomposition approach include ade4, amap, and psych, each one operating on a different definition of covariance matrix.

Specifically, the function ade4::dudi.pca (Thioulouse et al. 2018) is equivalent to running princomp(X, fix_sign = F) in base-R: both options generate the same covariance matrix, scores and loadings. Conversely, the function amap::acp (Lucas 2022) computes the eigenvalues and eigenvectors of $$\textbf{Y}^T\textbf{Y}$$. Whilst the same eigenvectors would have been obtained form the covariance matrix $$\textbf{S}$$ as defined in Sect. 3.2, the eigenvalues differ (e.g., Jolliffe 1986, Ch. 3). Confusingly, the output acp()$eig does not return these eigenvalues, but their square roots.

Unlike the previous functions, psych::principal (Revelle 2024) operates on a covariance matrix that corresponds to $$\textbf{S}$$. Yet it produces scores and loadings that are scaled—respectively, divided and multiplied—by acp()$eig. This type of implicit scaling, whilst not uncommon (see e.g., Gewers et al. 2021), may elude the user.

With regards to biplots, the packages considered here pose similar challenges as those encountered in the previous section. Functions like dudi.pca::scatter, acp::plot combine a plot of scores and a plot of loadings. Alternatively, one could implement ggbiplot with inputs generated by dudi.pca. Conversely, psych::biplot.psych leverages the implicit normalisation of scores and loadings discussed earlier, which is reminiscent of Eqs. 11, 12 except that it disagrees with Eq. 7.

The only specialised biplot package in this goup is BiplotGUI (la Grange et al. 2009): unlike other R packages examined here, it generates a self-contained, fully interactive user interface, with navigation menus and the option to export some of the outputs. Under the hood, BiplotGUI implements the concept of calibrated biplot axes (Gower et al. 2011). It precedes biplotEZ (Sect. 4.3), but shares some of the underlying logic. Unlike biplotEZ the elements of the diagonal matrix $\mathbf {\Delta }$ are the square roots of the eigenvalues of $\textbf{Y}^T\textbf{Y}$, which appears more correct although these are still divided by $\sqrt{n-1}$. As in biplotEZ, disagreement with Eq. 7 generates a discrepancy between the elements of $\mathbf {\Delta }$ and the singular values. The way the coordinates of a PC biplot are computed in BiplotGUI is similar to biplotEZ and unlike Eqs. 11, 12.

### Group 3: generalised SVD

The remaining 20% of implementations considered here are characterised by a specific approach to matrix decomposition, referred to as ‘generalised’ or ‘weighted’ SVD (see e.g., Abdi and Williams 2010; Greenacre 2010). Based on Fig. 1, popular packages in this group are FactoMineR (Lê et al. 2008), and vegan (Oksanen et al. 2025), both of which are ‘generalist’ in nature. The only specialised package implementing this approach is PCAmixdata (Chavent et al. 2022), which places a greater emphasis on the simultaneous handling of quantitative and categorical data within PCA.

**Fig. 5:**
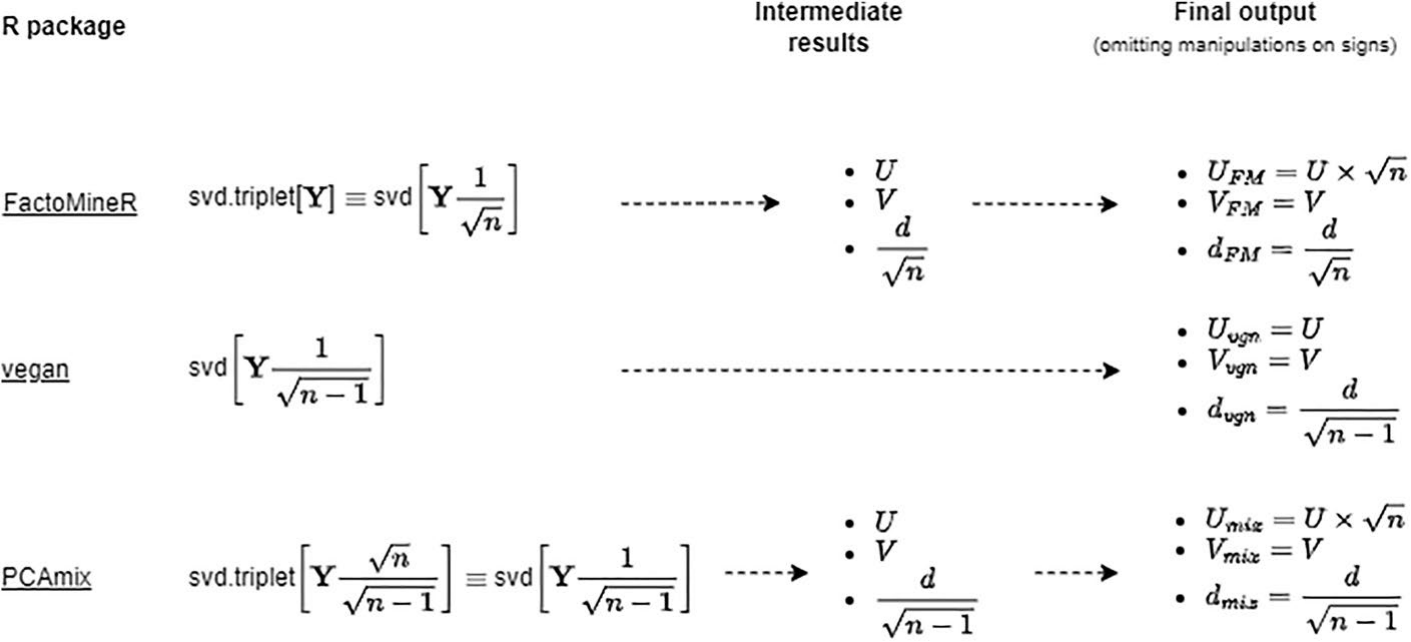
Schematic representation of how generalised SVD operates in selected R packages

In practice, a generalised SVD amounts to a conventional SVD of a weighted version of the centred data-matrix $\textbf{Y}$. Table 4 reports, under the “decomposition” column, the weighting schemes that reproduce the behaviour of specific implementations. Yet this equivalence is not immediately evident when examining functions like FactoMineR::svd.triplet. To bring clarity, Fig. 5 provides a schematic representation of how this approach operates in the implementations considered here.

In the case of FactoMineR, Fig. 5 shows that the generalised SVD affects both $\textbf{U}$ and $\textbf{D}$: unlike Eq. 6, these matrices are, respectively, multiplied and divided by $\sqrt{n}$. In practice, signs may also vary due to further manipulations occurring in svd.triplet. With this decomposition, the function FactoMineR::PCA returns a ‘hybrid’ set of biplot coordinates—neither a PC biplot, nor a scores and loadings biplot. These coordinates assume a modified version of Eq. 8, where both exponents $\alpha$ and $1-\alpha$ are replaced with 1, so that (1) the observations’ coordinates are simply the scores i.e., $\textbf{A}=\textbf{U}n^{1/2}\textbf{D}n^{-1/2}=\textbf{Z}$; whereas (2) the matrix of features coordinates is akin to a PC biplot, since $\textbf{B}=\textbf{V}\textbf{D}n^{-1/2}$.

The biplot coordinates produced by FactoMineR obviously disagree with Eqs. 12, 11 and therefore depart from our evaluation grid. Yet it is not uncommon to encounter similar ‘hybrid’ approaches in practice. An example is the so-called “HJ biplot” (Nieto et al. 2014), which may be implemented with packages like bpca.

The function vegan::rda carries out an SVD of $\textbf{Y}\frac{1}{\sqrt{n-1}}$. As Fig. 5 shows, the resulting singular values are equivalent to $\sqrt{\lambda _k}$ based on Eq. 7 ($k=1,\dots ,m$). Unlike most implementations, there is no obvious counterpart to the notion of PCA scores. Yet some of the outputs generated by vegan::scores, called sites and species, correspond to a scaled version of the PC biplot coordinates in Eqs.11, 12. Specifically: $\textbf{A}=\textbf{U}\psi$ and $\textbf{B}=\textbf{V}\textbf{D} /\psi$, where $\psi =\left( \sqrt{n-1}\sum _{k=1}^{m} \lambda _k\right) ^{1/2}$. The rationale for this specific additional scaling, however, is not immediately clear from the original script.

The last implementation in this group is the function PCAmixdata::PCAmix, which exhibits some aspects of FactoMineR and vegan, yet differs from both. Unlike other implementations, PCAmix imposes that the raw data matrix is always standardised, an aspect that contributes to the discrepancies in Tables 4, 5, 6. Assuming standardisation is omitted, Fig. 5 shows that a generalised SVD of $\textbf{Y}\left[ n/(n-1)\right] ^{1/2}$ is analogous to a conventional SVD of $\textbf{Y}\frac{1}{\sqrt{n-1}}$, except that $\textbf{U}$ is multiplied by $\sqrt{n}$. With these inputs, the PC biplot coordinates are generated following a ‘hybrid’ approach similar to FactoMineR’s, which yields slightly different outputs: $\textbf{A}=\textbf{Z}\left[ n/(n-1)\right] ^{1/2}$ and $\textbf{B}=\textbf{V}\textbf{D}(n-1)^{-1/2}$.

## Discussion

This section summarises key insights that emerge across the groups of implementations examined earlier. The discussion is structured around three areas: (1) methodological insights based on the proposed evaluation framework; (2) insights from published applications, beyond the specialised software routines reviewed; and (3) implications for practitioners based on insights from the case example.

### Methodological insights

At the start of this paper we set out to examine key computational aspects underpinning the implementations of PCA and biplots in R—an endeavour that differs substantially from running pre-built code ‘as is’ to assess e.g., how fast it executes.

Throughout the paper we outlined a ‘software-agnostic’ evaluation grid and compared a representative subset of PCA and biplots implementations in R against it. This constitutes a major departure from work providing a descriptive overview of alternative software options (e.g. Nieto et al. 2014; la Grange et al. 2009). Specifically, the ‘back-to-basics’ perspective adopted in this work emphasised the need to look under the hood and assess whether and to what extent specific methodological building blocks were taken into account by the implementations reviewed.

Our analysis suggests that departures from the identified building blocks are often due to computational choices that are routinely made under the hood, and are considered inconsequential. Nearly all pre-built implementations violate the fundamental relationships between scores variances, eigenvalues and singular values outlined in Eq. 5 and Eq. 7 (e.g., singular values and score’s St.Dev. are used interchangeably). Whilst these identities are often assumed, they do not follow without caveats from the outputs of most implementations. The prevalence of SVD-based approaches may obscure the rationale that underpins the concepts of *scores* and *loadings* discussed in Sect. 3.2 and generate confusion about singular values and eigenvalues.

The distinction between PC biplots and what we called “scores and loadings” biplots appears underplayed in practice, despite being unambiguously rooted in the joint computation of features’ and observations’ coordinates (Eq. 11, 12). This is all the more relevant considering that the chosen computational strategy determines which properties hold for the geometrical representations that result from it, and the ability to convey reliable information about the underlying data.

It is surprising that none of the specialised biplot implementations reviewed in Table 4, 5, 6 succeeds in matching exactly the set of coordinates that one would expect from a software-agnostic understanding of the concept of PC biplot. As a consequence, most properties that are commonly assumed to hold could not be verified easily. Some packages e.g., bpca appear to have strong fundamentals, but impose additional processing steps that ultimately alter some of the key relationships considered in our grid.

Simpler, base-R implementations such as prcomp and biplot appear to be most convincingly aligned with the rationale leading to Eqs. 11, 12, despite not being immune from imprecisions. By contrast, functions with a sophisticated computational infrastructure and richer visualisation capabilities—hence popular amongst users—may be harder to reconcile with a software-agnostic understanding of PCA and biplots.

### Insights from published applications

To broaden the discussion beyond specialised software routines, we also touch on how PCA and biplots may be jointly implemented in practice on the basis of a small selection of 15 academic papers from methodology-oriented, multidisciplinary journals including *Quality & Quantity*—see Table 10.

**Table 10:** Categorised references (chronological order)

Reference	Primary		Computation			Software		Matrix	
	technique		detail			used		decomposition.	
	PCA	other	PCA	Biplot	other	R	other	Eigenvalue	SVD
Fernández-Aguirre et al. (2014)		$\bullet$			$\bullet$	$\bullet$		$\bullet$	
Scippacercola et al. (2019)		$\bullet$			$\bullet$				
Amenta et al. (2019)		$\bullet$			$\bullet$			$\bullet$	
Ciommi et al. (2019)	$\bullet$								
Zambon et al. (2020)	$\bullet$								
Guardabascio et al. (2023)	$\bullet$		$\bullet$			$\bullet$		$\bullet$	
Alaimo et al. (2023)		$\bullet$			$\bullet$				
De Falco and Irpino (2024)		$\bullet$	$\bullet$			$\bullet$			$\bullet$
Cova et al. (2017)	$\bullet$		$\bullet$			$\bullet$		$\bullet$	
Zhang and Ding (2023)	$\bullet$					$\bullet$			
Abascal and de Rada (2014)		$\bullet$							
Carlos Lozares and Muntanyola (2015)	$\bullet$								
Abascal and Landaluce (2018)		$\bullet$					$\bullet$		
Landaluce-Calvo et al. (2022)	$\bullet$						$\bullet$		
Durose and Dean (2023)		$\bullet$							

PCA and biplots are so well-established and readily implemented that seldom is it required of a practical implementation to reference their computational aspects explicitly: this applies to over 85% of the reviewed references with regards to PCA—100% when it comes to biplots or equivalent graphical representations.

The few examples that provide mathematical context include Guardabascio et al. (2023) and De Falco and Irpino (2024): the former assumes an eigen-decomposition approach, the latter a (weighted) SVD. Other work may disclose some computations, but not to an extent that reveals the stance taken on the methodological issues discussed earlier (e.g., Cova et al. 2017). Some work refers to PCA as a benchmark whilst providing more details about other techniques (e.g., Fernández-Aguirre et al. 2014).

Although most computational aspects are left to pre-built software routines, only 40% of the reviewed references mention these tools explicitly, whereas 33% rely on R implementations without specifying which function or library is deployed.

### Implications for practitioners

There is no shortage of accessible resources at the analyst’s fingertips when it comes to implementing PCA and PC biplots. Yet the previous discussion suggests there is also room for methodological ambiguity, and little incentive to look under the hood.

Against this backdrop, we provide practitioners seeking greater methodological clarity with a parsimonious framework—summarised in Table 3—which may help ascertain whether a software routine “does what it says on the tin”, as it were, beyond self-reported intents and capabilities. We are not aware of other resources providing this type of guidance—including dedicated monographies (e.g., Gower et al. 2011).

Rather than providing a descriptive overview, we put our own framework and 15 pre-built implementations to work against the same numerical example based on real-world data on clinical trial supply chains. Tables 4, 5, 6 provide a concise reference for verifying commonly held assumptions about, and properties of, PC biplots.

Those expecting drastic discrepancies between implementations based on eyeball inspection of their graphical displays alone might feel disappointed. Yet for implementations underpinned by the same methods, discrepancies often arise at a deeper analytical level. The proposed checks and balances could help mitigate common biases in the perception of data represented through a single visual idiom—which is a pressing issue in managerial decision-making (Bendoly and Clark 2017, Ch. 1 &3).

Regardless of the limitations found in the reviewed implementations, it is worth emphasising that it is a strength, not a weakness, of open-source implementations to allow and promote a level of scrutiny and reproducibility. The depth of analysis this work is based on would not be possible for a commercial, off-the-shelf equivalent.

## Closing remarks

In a context where implementing PCA and biplots has become a conditioned reflex, facilitated by pre-built software routines, one can hardly resist the temptation to take their internal workings for granted. To counter that, this research proposed a back-to-basics comparison of PCA and biplots implementations in base R and a selection of contributed R packages. The approach highlighted useful equivalences that should hold if the computational rationale underpinning each technique is followed correctly.

Findings suggest that discrepancies from a software-agnostic understanding of PCA and biplots do arise, in both base R and contributed R packages, from seemingly innocuous computational choices made under the hood. Surprisingly, the identified verification equivalences rarely follow without caveats from the output of specific implementations alone. What is more, biplots are often just a misnomer due to imprecisions in how the underlying computational aspects are dealt with.

This research makes no pretence of comprehensiveness, and several implementations of potential interest could not be included. Specifically, more advanced computational approaches were deemed out of scope considering our emphasis on getting back to basics. Our intent is not so much to direct practitioners towards a ‘best overall' implementation, as an invitation to adopt a software-agnostic perspective.

Despite its limitations, the hoped for outcome of this note is to elevate aspects that are usually disregarded for comparative purposes, and to address discrepancies that users may find elusive unless they are prepared to take a closer look under the hood of their implementation of choice.

## Appendix A: Calibrated biplot axis approach

In Sect. 3.3 we touched on the “calibrated biplot axes” approach adopted by several R packages. Here we illustrate its rationale following Gower et al. (2011, Ch. 2).

A key assumption is that the coordinates for observations and features are obtained from Eq. 8 choosing $\alpha = 1$, from which we get the rank-2 approximation (Eq. 9):

A1 $$\begin{aligned} \textbf{Y} \approx \textbf{Y}_{(2)}=\textbf{Z}_{(2)}\textbf{V}^T_{(2)} \end{aligned}$$

where only the first two columns of $\textbf{Z}$ and $\textbf{V}$ are retained for display. For a given observation *i* and feature *j* Eq. A1 is just the inner product between the *i*-th row of the scores matrix, $\textbf{z}_{i \bullet }$ and the *j*-th ow of the loadings matrix, $\textbf{v}_{j \bullet }$:

A2 $$\begin{aligned} y_{ij} \approx \langle { \textbf{z}_{i \bullet },\textbf{v}_{j \bullet } }\rangle \end{aligned}$$

Invoking Eq. 15 we obtain what is often referred to as “calibration marker”:

A3 $$\begin{aligned} \langle { \textbf{z}_{i \bullet },\textbf{v}_{j \bullet } }\rangle&=\cos \theta _{i,j} \cdot \left\Vert \textbf{z}_{i \bullet } \right\Vert _2 \cdot \left\Vert \textbf{v}_{j \bullet } \right\Vert _2 \nonumber \\&= \mu \end{aligned}$$

Gower et al. (2011, p. 23) suggest that the inner product in Eq. A3 should produce the same scalar $\mu$ if $\textbf{z}_{i \bullet }$ is replaced by the coordinates of any point along the line connecting point *i* to its orthogonal projection onto the feature vector *j*.

One such point is the projection itself, whose unknown coordinates can be expressed as some proportion $\lambda$ of feature *j*’s original coordinates, leading to:

A4 $$\begin{aligned} \langle {\lambda \textbf{v}_{j \bullet },\textbf{v}_{j \bullet } }\rangle&=\mu \nonumber \\ \lambda&= \frac{\mu }{\left\Vert \textbf{v}_{j \bullet } \right\Vert _2^2} \end{aligned}$$

With Eq. A4, one calibrates the biplot axis corresponding to feature *j* by setting a range of values for $\mu$ corresponding to the marks one wishes to display on the axis. For each $\mu$, one obtains $\lambda$ from Eq. A4. The position of the marker onto the features’ axis is then given by $\lambda \textbf{v}_{i \bullet }$ in terms of the feature’s original coordinates.

## Appendix B: List of online supplements

The following complementary materials are provided as online supplements:

- A self contained, thoroughly annotated R script to reproduce the examples, analysis and code listings in Sects. 3.1 and 4. Available on GitHub at: https://github.com/Dr-Eti/YAPCABIC.git
- A spreadsheet with the full list of R implementations referred to in Section 2. Also available on GitHub at: https://github.com/Dr-Eti/YAPCABIC/blob/main/R_PCA_pkgs_list.xlsx

## Footnotes

1 I am indebted to Dr. S. Dray for pointing out this distinction in their comments on an earlier draft.

2 Several online resources provide useful animations (e.g., Starmer 2018; amoeba 2015)
